# Sarcopenia Predicts Postoperative Cognitive Impairment and Poor Surgical Outcomes in Older Adults: A Prospective Cohort Study

**DOI:** 10.1002/jcsm.70346

**Published:** 2026-07-15

**Authors:** Xingming Tang, Yuncai Gu, Li Wang, Xiaochu Wu, Yipu Ren, Shudie Zhou, Yulong Cai, Xian Du, Xun Xia, Tianyao Zhang

**Affiliations:** ^1^ Department of Anesthesiology The First Affiliated Hospital of Chengdu Medical College Chengdu China; ^2^ School of Clinical Medicine Chengdu Medical College Chengdu China; ^3^ West China School of Public Health and West China Fourth Hospital, Sichuan University Chengdu China; ^4^ National Clinical Research Center for Geriatrics West China Hospital, Sichuan University Chengdu China

**Keywords:** acute postoperative cognitive impairment, fall, older adult patients, postoperative complications, sarcopenia

## Abstract

**Background:**

Sarcopenia is a progressive skeletal muscle disorder prevalent in older adults, yet its role as a risk factor for acute postoperative cognitive decline—an early manifestation within the spectrum of perioperative neurocognitive disorders (PND)—remains underexplored. We hypothesize that preoperative sarcopenia increases the incidence of early postoperative cognitive impairment and adverse surgical outcomes in geriatric patients.

**Methods:**

This prospective cohort study enrolled 443 older adult patients (mean age 72.8 ± 5.8 years, 58.3% male) undergoing elective noncardiac surgery at a single centre in China. Preoperative sarcopenia was diagnosed according to the 2019 Asian Working Group for Sarcopenia (AWGS) criteria, which included assessments of muscle mass, strength, and physical performance. Neurocognitive function was assessed via the Mini‐Mental State Examination (MMSE) 1 day before and 3 days after surgery, with acute cognitive decline defined as a postoperative decrease of ≥ 2 points. Of the 443 patients, 391 (88.2%) completed 6‐month telephone follow‐up for assessment of longer‐term functional outcomes. In an exploratory subset of 60 patients, preoperative faecal samples underwent 16S rRNA sequencing and untargeted metabolomics to characterize gut microbial and metabolic signatures.

**Results:**

The prevalence of preoperative sarcopenia was 28.0% (124/443). Acute postoperative cognitive decline occurred in 27.3% (121/443) of patients. Multivariate logistic regression identified preoperative sarcopenia (adjusted OR = 3.291; 95% CI: 1.295–7.531; *p* < 0.001) and frailty (adjusted OR = 4.012; 95% CI: 1.854–8.456; *p* < 0.001) as independent risk factors for acute cognitive decline. Sarcopenic patients exhibited significantly higher rates of postoperative complications (adjusted RR = 1.21; 95% CI: 1.12–1.44; *p* = 0.025) and ICU admission (adjusted RR = 2.41; 95% CI: 1.03–4.41; *p* = 0.008). Among the 391 patients with complete 6‐month follow‐up, the sarcopenia group exhibited elevated risks of falls (adjusted RR = 2.89; 95% CI: 1.55–5.08; *p* = 0.028) and all‐cause mortality (adjusted RR = 3.07; 95% CI: 1.17–7.87; *p* = 0.016). Exploratory microbiome analysis revealed an elevated Firmicutes/Bacteroidetes ratio, reduced Bacteroides abundance, and upregulated faecal stercobilin and estradiol derivatives in sarcopenic patients who developed cognitive decline.

**Conclusion:**

Preoperative sarcopenia is an independent risk factor for acute postoperative cognitive impairment and poor surgical outcomes in older adults. These findings support the integration of sarcopenia screening into preoperative risk stratification and suggest a potential role of the gut–muscle–brain axis in perioperative neurocognitive vulnerability.

## 1 Introduction

According to the United Nations World Population Prospects Report, the proportion of individuals aged ≥ 60 years is projected to rise from 13.5% in 2020 to 22% by 2050. As the population ages, the proportion of older adult patients undergoing surgery will also rise significantly. In the United States, older adult patients account for up to 50% of all surgical patients, representing 30% in China [1] Older adult surgical patients confront a complex array of perioperative challenges encompassing age‐related physiological decline, heightened oxidative stress, nutritional deficiencies, progressive cognitive impairment and psychosocial vulnerability. These multifactorial comorbidities collectively contribute to increased surgical risk and compromised postoperative recovery trajectories [2]. Despite advances in surgical procedures and perioperative care, many older adult patients still suffer from cognitive decline, postoperative morbidity, prolonged hospital stays, increased healthcare costs and reduced quality of life [3]. Identifying modifiable risk factors and implementing supportive interventions are crucial for improving postoperative cognitive outcomes in this vulnerable population.

The term perioperative neurocognitive disorders serves as an overarching descriptor for cognitive impairment occurring across the perioperative continuum, encompassing preoperative deficits, acute postoperative delirium (POD), delayed neurocognitive recovery (within 30 days) and long‐term postoperative cognitive dysfunction (POCD) up to 12 months post‐surgery [4]. Although the long‐term consequences of PND are profound, the immediate postoperative period represents a critical window of vulnerability wherein acute perturbations in cognition—primarily manifesting as delirium or early cognitive decline—can precipitate a cascade of adverse events and functional decline [5]. Accordingly, the present study focuses on the acute manifestation of this spectrum, specifically examining the impact of preoperative sarcopenia on early postoperative cognitive decline occurring within the first 3 days following surgery.

Multiple risk factors contribute to the development of PND, including advanced age, education level, type of surgery, preoperative comorbidities, anaesthesia methods and intraoperative events such as hypoxia and hypotension [6]. Among these, advanced age is widely recognized as an independent risk factor [7]. With the growing focus on the older adult population, researchers have identified that muscle loss is particularly severe in this group [8].

Sarcopenia, an age‐related syndrome characterized by a decline in muscle mass and strength, may or may not be accompanied by physical disorders [9]. Muscle mass typically peaks around the age of 30 years and gradually declines with age. The global prevalence of sarcopenia is estimated to be between 6% and 12%, with the incidence rate among the older adult population over 65 years old reaching up to 33% [10]. Clinical studies have demonstrated that patients with low muscle mass are at a higher risk of postoperative complications, prolonged hospital stays and increased incidence of postoperative delirium [11, 12].

Research has identified a regulatory loop between the brain and skeletal muscle mediated by myokines, known as the muscle–brain axis. Myokines, such as brain‐derived neurotrophic factor (BDNF), insulin‐like growth factor and interleukin‐6 secreted by skeletal muscles, regulate neuronal proliferation and differentiation through specific molecular pathways, thereby influencing cognitive function [13]. An animal study further revealed that muscle atrophy in the hindlimbs of rats is associated with a higher incidence of PND [14].

Given the existing gaps in understanding, further research is warranted to explore the correlation between preoperative sarcopenia and postoperative cognitive outcomes in older adult patients. To address this, we designed a prospective cohort study to investigate the impact of preoperative sarcopenia on acute postoperative cognitive decline—an early manifestation within the PND spectrum—as well as its effects on short‐term surgical complications and long‐term functional outcomes including falls and mortality, in this vulnerable population.

## 2 Methods

The clinical study protocol is in accordance with the Declaration of Helsinki. This work has been reported in line with the STROCSS criteria and was registered in the Chinese Clinical Trial Registry (ChiCTR 2200065347) [15]. Ethics approval was obtained from the Ethics Committee of The First Affiliated Hospital of Chengdu Medical College (2021CYFYIRB‐BA‐67‐01). Informed consent was obtained from all participants.

The protocol was written in accordance with the Standard Protocol Items: Recommendations for Interventional Trials guidelines. This is a single‐centre, prospective, observational cohort study of preoperative sarcopenia and postoperative cognitive outcomes in geriatric patients undergoing noncardiac surgery. Therefore, the study was designed without intervention, randomization and blindness.

### 2.1 Study Participants

This prospective cohort study enrolled 480 patients aged ≥ 65 years classified as American Society of Anesthesiologists (ASA) physical status II–III, who were scheduled to undergo noncardiac surgical procedures at the First Affiliated Hospital of Chengdu Medical College during the study period from 1 March 2022 to 30 April 2023. Patients were excluded if they met any of the following criteria: (1) refusal by the patient or their family to sign the informed consent form; (2) presence of systemic diseases affecting the heart, lungs, liver, kidneys or other major organs; (3) presence of central nervous system disorders, neurological conditions, or mental illnesses; (4) history of drug or alcohol dependence; (5) inability to complete gait speed and grip strength assessments; (6) visual, hearing or communication impairments that hindered their ability to complete questionnaires or cooperate with examinations; (7) presence of missing limbs, cardiac pacemakers, metal stents or other implants that precluded body composition analysis. The trial would be terminated if any of the following occurred: (1) the participant chose to withdraw from the study at any point; (2) the occurrence of significant intraoperative events such as massive haemorrhage, severe allergic reactions, severe cardiovascular or cerebrovascular accidents, or cardiac arrest.

### 2.2 Definitions of Sarcopenia

The appendicular lean mass index (ASMI) was assessed using bioelectric impedance analysis (BIA) to evaluate skeletal muscle mass, 1 day before surgery. Low ASMI was diagnosed as less than 7.0 kg/m^2^ for men or less than 5.7 kg/m^2^ for women. Muscle strength was measured by handgrip strength. It was measured using a Xiangshan Spring Grip Strength Dynamometer, with the highest value recorded from two trials of maximum‐effort isometric contraction of the dominant hand. Low muscle strength was defined as a grip strength of less than 18 kg in women or less than 28 kg in men [16]. Physical performance was evaluated by measuring the time taken to walk 6 m at a normal pace from a moving start, without deceleration. The average of at least two trials was recorded, with low physical performance defined as a walking speed of less than 1.0 m/s over 6 m [16].

### 2.3 Neurocognitive Evaluation

To assess the patients' neurocognitive levels, a well‐trained investigator administered the MMSE 1 day before and 3 days after surgery [17]. If the patient's MMSE score decreased by 2 or more points 3 days post‐surgery compared with their preoperative score, it was considered indicative of postoperative cognitive decline, signifying the occurrence of APCI [18]. Of note, while the 2018 Nomenclature consensus defines PND as a spectrum including both POD and longer‐term cognitive dysfunction, our study focused specifically on early acute cognitive decline assessed on postoperative Day 3. Given the timing and the use of the MMSE, which assesses cognitive performance but not attentional fluctuations or level of consciousness, the observed condition in this cohort aligns most closely with the clinical domain of POD or acute encephalopathy, rather than persistent POCD.

### 2.4 Clinical Data Collection

Demographic characteristics and clinical data were collected from each participant. Frailty was assessed using the FRAIL scale, the nutritional status was assessed using the Mini Nutritional Assessment (MNA) [19], and the assessment of functional status uses the Activities of Daily Living (ADL) scale [20].

During surgery, data on intraoperative conditions, including the type of surgery, anaesthesia method, duration of surgery, duration of anaesthesia, intraoperative fluid replacement and blood loss, were recorded. Postoperative data collection included the occurrence of APCI, complications, ICU outcomes and length of hospital stay. Postoperative complications were diagnosed based on the Clavien–Dindo classification system. Additionally, follow‐up via telephone was conducted to gather information on falls, re‐admission and mortality status 6 months after discharge.

### 2.5 Outcome Measures

The primary outcome was the incidence of APCI, the third day after surgery. Secondary outcomes included: (1) the incidence of postoperative complications during hospitalization, ICU admission rate, and length of hospital stay; (2) the unplanned readmission rate within 6 months after discharge; (3) the fall rate within 6 months after discharge; (4) all‐cause mortality within 6 months after discharge.

### 2.6 Quality Control Measures

#### 2.6.1 Standardization of Assessment Tools

Neurocognitive function was assessed using the standardized MMSE scale. Preoperative assessments were conducted in quiet wards 1 day before surgery, while postoperative assessments were performed 3 days postoperatively in quiet wards when patients were awake with stable vital signs, avoiding interference from sedative medications. Every 3 months, neuropsychology experts conducted on‐site random checks of assessment procedures to ensure implementation consistency.

#### 2.6.2 Personnel Training

Assessors received standardized MMSE evaluator training including simulated practical tests. Retraining sessions were conducted every 6 months to maintain assessment consistency.

#### 2.6.3 Exploratory Gut Microbiome and Metabolomics Sub‐Study

To explore potential biological mechanisms linking preoperative sarcopenia to acute postoperative cognitive decline, a subset of consecutively enrolled patients (*n* = 60) provided preoperative faecal samples. Samples were collected 1 day before surgery, immediately frozen at −80°C and transported on dry ice for analysis. 16S rRNA gene sequencing was performed to characterize gut microbial composition, and untargeted metabolomics was conducted to profile faecal metabolites. Detailed protocols for DNA extraction, library preparation, sequencing and bioinformatics analysis are provided in Supporting Information S1.

### 2.7 Sample Size

This study is a prospective cohort study designed to investigate the predictive effect of preoperative sarcopenia on postoperative neurocognitive disorders. Based on preliminary experimental data (*n* = 149), the incidence of sarcopenia was 22.14% (33/149). The incidence of postoperative neurocognitive disorders was 42.42% (14/33) in the sarcopenia group and 19.82% (23/116) in the non‐sarcopenia group, with an odds ratio (OR) of 2.75 between the two groups. Using PASS 15.0 software for sample size calculation based on this effect size: with *α* = 0.05, *β* = 0.10, and selecting a two‐independent sample proportion test; using the postoperative neurocognitive disorder incidence rates of 42.42% (p_1_) in the sarcopenia group and 19.82% (p_2_) in the non‐sarcopenia group as benchmarks, the software calculated a required total sample size of 335 cases (67 in the Sar group and 268 in the non‐Sar group, exposure ratio 1:4). Conservatively allowing for a 20% dropout buffer, the final determined sample size includes 89 cases in the sarcopenia group and 313 cases in the non‐sarcopenia group, totalling 402 cases.

### 2.8 Statistical Analysis

The distribution of variables was assessed using the Shapiro–Wilk test. Continuous variables that followed a normal distribution were presented as mean ± standard deviation (SD) and compared using Student's *t*‐test. For non‐normally distributed continuous variables, data were reported as median (interquartile range, IQR) and comparisons were made using the Mann–Whitney *U* test. Categorical variables were expressed as frequencies (%) and were analysed using the chi‐squared test or Fisher's exact test.

The identified independent risk factors associated with APCI, variables that showed statistical significance (*p* < 0.05) in the initial group comparisons, as well as those with a *p*‐value < 0.1 in univariate logistic regression analysis, were included in a multivariate regression model. Results were presented as adjusted odds ratios (OR) with 95% confidence intervals (CI).

Modified Poisson regression was employed to estimate the risk ratio (RR) of adverse outcomes (including APCI, postoperative complications, ICU admission, falls and mortality) between the sarcopenia and non‐sarcopenia groups, adjusting for potential confounders. Results were presented as adjusted risk ratios (RR) and 95% CI.

To preliminarily assess the potential impact of sarcopenia on long‐term prognosis, we performed an exploratory Kaplan–Meier survival analysis. The endpoint event was all‐cause death within 6 months after discharge, and survival time was defined as the time (in months) from hospital discharge to death or the last follow‐up. And differences between groups were compared using the log‐rank test.

All statistical analyses were performed using IBM SPSS Statistics version 26.0 (IBM Corp., Armonk, NY, USA) and survival and survminer packages in R software (version 4.x). A two‐sided *p*‐value < 0.05 was considered statistically significant.

## 3 Result

From March 2022 to April 2023, this study recruited a total of 505 older adult patients aged ≥ 65 years who were scheduled for elective noncardiac surgery. During the preoperative assessment process, 10 patients refused to sign the informed consent form, 5 were unable to complete the sarcopenia assessment, 4 were unable to complete the preoperative cognitive function assessment, 2 had severe systemic diseases such as cardiopulmonary, hepatic or renal diseases, 2 had severe drug or alcohol dependency and 2 were unable to complete other questionnaires. Due to these reasons, they were excluded from the study, leaving 480 patients who completed the preoperative assessment. Among them, 14 patients were excluded due to incomplete postoperative cognitive function assessment, 13 had their preoperative surgery cancelled, 1 experienced intraoperative cardiac arrest, 3 had severe arrhythmias/hypotension during surgery, and 3 withdrew from the study at this stage. Consequently, a total of 443 patients completed both preoperative and postoperative assessments. We meticulously documented the preoperative and postoperative MMSE scores for each patient, with the specific trends illustrated in Figure S1. The data from these patients can be incorporated into the statistical analysis for the first‐phase study on risk factors for APCI. During the 6‐month follow‐up period, 37 patients were lost to follow‐up, and 15 withdrew from the study at this stage. Ultimately, 391 patients could be included in the statistical analysis for the second‐phase study on the prognosis of the Sar group versus the N‐Sar group (Figure S2).

A total of 121 patients (27.3%) met the criteria for acute postoperative cognitive decline (APCI group). Baseline demographic and clinical characteristics are summarized in Table 1. Compared with the N‐APCI group, patients who developed acute cognitive decline were significantly older, more likely to be frail and had a markedly higher prevalence of preoperative sarcopenia (all *p* < 0.05). Groups were otherwise well balanced with respect to sex, BMI, education level and comorbidities such as hypertension and diabetes (Table 1).

**TABLE 1:** Demographic and disease characteristics of acute postoperative cognitive impairment group and not acute postoperative cognitive impairment group.

Baseline characteristics	Total cohort *n* = 443 (%)^a^	APCI group *n* = 121 (%)^a^	N‐APCI group *n* = 322 (%)^a^	*p*
Age	72 (68–77)	73 (69–78)	71 (68–75)	0.036*
Weight, kg	58.2 ± 7.9	57.4 ± 6.9	58.4 ± 8.9	0.301
BMI, kg/m^2^ ^b^	22.9 ± 3.3	23.2 ± 3.2	22.9 ± 3.5	0.389
Gender				0.327
Male	336 (75.48)	93 (76.86)	243 (75.47)	
Female	107 (24.16)	28 (23.14)	79 (24.53)	
Education level				0.814
Illiterate	72 (16.25)	17 (14.05)	55 (17.08)	
Primary	223 (50.34)	66 (54.55)	157 (34.09)	
Junior	91 (20.54)	22 (18.18)	69 (15.58)	
High/vocational	42 (9.27)	12 (9.92)	30 (6.77)	
University/college	15 (3.39)	4 (3.31)	11 (2.48)	
MMSE, score	26 (24–28)	26 (24–28)	26 (24–28)	0.154
Pre‐NCD				0.037*
Yes	87 (19.6)	31 (25.6)	56 (17.4)	
No	356 (80.4)	90 (74.4)	266 (82.6)	
Frailty				< 0.001***
Yes	163 (36.8)	71 (58.7)	92 (28.6)	
No	280 (63.2)	50 (41.3)	230 (71.4)	
Degree of frailty				< 0.001***
Non‐frail	280 (63.2)	50 (41.3)	230 (71.4)	
Pre‐frail	87 (19.6)	40 (33.1)	47 (14.6)	
Frail	76 (17.2)	31 (25.6)	45 (14.0)	
Hypertension				0.793
Yes	185 (41.8)	58 (47.9)	127 (39.4)	
No	258 (58.2)	63 (52.1)	195 (60.6)	
Diabetes				0.448
Yes	122 (27.5)	37 (30.6)	85 (26.4)	
No	321 (72.5)	84 (69.4)	237 (73.6)	
Sarcopenia				< 0.001***
Yes	124 (28.0)	52 (43.0)	72 (22.4)	
No	319 (72.0)	69 (57.0)	250 (77.6)	

*Note:* The APCI group refers to the group with acute postoperative cognitive impairment. The N‐APCI group refers to the group without acute postoperative cognitive impairment.

Abbreviations: APCI, acute postoperative cognitive impairment; BMI, body mass index; MMSE, Mini‐Mental State Examination; pre‐NCD, pre‐existing neurocognitive disorders.

^a^ Values are reported as *n* (%) unless otherwise indicated.

^b^ Calculated as weight in kilograms divided by height in meters squared.

* *p* < 0.05.

** *p* < 0.01.

*** *p* < 0.001.

### 3.1 Consistence in Intraoperative Conditions

There were no significant differences between the two groups concerning anaesthesia methods, surgical procedures, use of dexmedetomidine, combined neural block anaesthesia, use of analgesia pumps, operation time, anaesthesia duration, fluid input or blood loss (*p* > 0.05, Table S1).

### 3.2 Consistence in Postoperative Outcomes

Postoperative outcomes, including the incidence of complications, ICU admission rates and length of hospital stay, showed no significant differences between the APCI and N‐APCI groups (*p* > 0.05, Table S2).

### 3.3 Preoperative Sarcopenia and Frailty as Independent Risk Factors for APCI

Univariate logistic regression analysis identified several factors associated with an increased risk of acute postoperative cognitive decline, including older age, preoperative frailty, pre‐NCD and sarcopenia. Complete univariate results for all candidate variables are presented in Table S3. Variables with a *p* < 0.1 in univariate analysis—namely, age, pre‐NCD, frailty, sarcopenia, hypertension, anaesthesia method and intraoperative dexmedetomidine infusion—were subsequently entered into a multivariate logistic regression model. After adjusting for these covariates, both preoperative frailty (adjusted OR = 4.012; 95% CI: 1.854–8.456; *p* < 0.001) and sarcopenia (adjusted OR = 3.291; 95% CI: 1.295–7.531; *p* < 0.001) remained independent predictors of acute postoperative cognitive decline (Table 2). No other variables retained statistical significance in the adjusted model. After 6 months of follow‐up, 391 participants completed the telephone interview. As detailed in Table 3, patients in the sarcopenia group exhibited significantly poorer preoperative functional and nutritional status, reflected by lower MMSE, MNA, and ADL scores, and a higher burden of frailty (all *p* < 0.001, Table 3).

**TABLE 2:** Multivariate regression analysis for risk factors of acute postoperative cognitive impairment.

Variables	OR	95% CI	*p*
Frailty	4.012	1.854–8.456	0.002**
Pre‐NCD	0.749	0.462–1.316	0.304
Dexmedetomidine	0.770	0.468–1.267	0304
Sarcopenia	3.291	1.295–7.531	0.006**
Anaesthesia method	0.695	0.428–1.129	0.952
Hypertension	1.447	0.911–1.068	0.124
Age	1.029	0.992–1.068	0.124

Abbreviations: CI, confidence interval; OR, odds ratio; pre‐NCD, pre‐existing neurocognitive disorders.

* *p* < 0.05.

** *p* < 0.01.

*** *p* < 0.001.

**TABLE 3:** Demographic and disease characteristics of the sarcopenia group and not sarcopenia group.

Clinical characteristics	Total cohort *n* = 391 (%)^a^	Sar group *n* = 106 (%)^a^	N‐Sar group *n* = 285 (%)^a^	*p*
Age, median (IQR), years	68 (72–77)	68 (73–77)	68 (71–76)	0.309
BMI, mean ± SD, kg/m^2^	22.99 ± 3.33	23.03 ± 3.11	22.95 ± 3.47	0.842
Gender				0.336
Male	222 (56.1)	56 (52.8)	166 (58.2)	
Female	169 (43.2)	50 (47.2)	119 (41.8)	
Education level				0.174
Illiterate	59 (15.1)	18 (17.0)	41 (14.4)	
Primary	207 (52.9)	51 (48.1)	156 (54.7)	
Junior	67 (17.1)	24 (22.6)	43 (15.1)	
High	39 (10.0)	10 (9.4)	29 (10.2)	
University	19 (4.9)	3 (2.9)	16 (5.6)	
MMSE, median (IQR), score	26 (24–28)	25 (23–26)	26 (25–28)	< 0.001***
Pre‐NCD				< 0.001***
Yes	84 (21.5)	30 (28.3)	54 (18.9)	
No	307 (78.5)	76 (71.7)	231 (81.1)	
MNA				< 0.001***
Normal	270 (69.1)	61 (57.6)	209 (73.3)	
Risk of malnutrition	64 (16.4)	19 (17.9)	45 (15.8)	
Malnutrition	57 (14.5)	26 (24.85)	31 (10.9)	
ADL				0.003**
Normal	336 (85.9)	84 (79.2)	252 (88.4)	
Minor disorders	26 (6.7)	11 (10.4)	15 (5.3)	
Moderate disorders	19 (4.9)	6 (5.7)	13 (4.6)	
Severe disorders	10 (2.5)	5 (4.7)	5 (1.7)	
Complete dependence	0 (0)	0 (0)	0 (0)	
Degree of frailty				< 0.001***
No frailty	218 (55.8)	50 (47.2)	168 (59.0)	
Pre‐frailty	90 (23.0)	25 (23.6)	65 (22.8)	
Frailty	83 (21.2)	31 (29.2)	52 (18.2)	
Hypertension				0.154
Yes	163 (41.7)	50 (47.2)	113 (39.7)	
No	228 (58.3)	56 (52.8)	172 (60.3)	
Diabetes				0.091
Yes	107 (27.4)	35 (33.0)	72 (25.3)	
No	285 (72.6)	71 (67.0)	213 (74.7)	

*Note:* The Sar group refers to the group with sarcopenia before surgery. The N‐Sar group refers to the group without sarcopenia before surgery.

Abbreviations: ADL, activities of daily living; BMI, body mass Index; IADLs, instrumental activities of daily living; MMSE, Mini‐Mental State Examination; MNA, Mini nutritional assessment; pre‐NCD, pre‐existing neurocognitive disorders.

^a^ Values are reported as *n* (%) unless otherwise indicated.

* *p* < 0.05.

** *p* < 0.01.

*** *p* < 0.001.

### 3.4 Consistency in Intraoperative Conditions Between Sar Group and N‐Sar Group

There was no significant difference between Sar group and N‐Sar group in terms of anaesthesia methods, surgical procedures, use of dexmedetomidine, combined neural block anaesthesia, use of analgesia pumps, operation time, anaesthesia duration, fluid input and blood loss (*p* > 0.05, Table 3).

### 3.5 Increased Incidence of APCI, ICU Admission and Postoperative Complications in the Sarcopenia Group

Among the 391 patients who underwent surgery, 119 (30.4%) developed APCI. The risk ratio (RR) of APCI in the Sar group was 1.88 (95% CI: 1.275–2.311, *p* < 0.001) compared with the N‐Sar group, with an adjusted RR of 1.65 (95% CI: 1.217–1.115, *p* < 0.001). The RR of ICU transfer in the Sar group was 2.66 (95% CI: 1.221–4.413, *p* = 0.005), with an adjusted RR of 2.41 (95% CI: 1.027–4.409, *p* = 0.008). The RR of postoperative complications in the Sar group was 1.39 (95% CI: 1.108–1.769, *p* = 0.016), with an adjusted RR of 1.24 (95% CI: 1.123–1.439, *p* = 0.025). There was no significant difference in the total length of hospitalization between the two groups (*p* > 0.05) as shown in Tables 4 and S5.

**TABLE 4:** Unadjusted RR and adjusted RR values for postoperative short term outcomes of the sarcopenia group and not sarcopenia group.

Short‐term outcomes	Sar group *n* = 106 (%)^a^	N‐Sar group *n* = 285 (%)^a^	Unadjusted RR (95% CI)	*p*	Adjusted RR (95% CI)	*p*
APCI			1.88 (1.275–2.311)	< 0.001***	1.65 (1.217–1.115)	< 0.001***
Yes	49 (46.3)	70 (24.6)				
No	57 (53.7)	215 (75.4)				
Transferred to ICU			2.66 (1.221–4.413)	0.005**	2.41 (1.027–4.409)	0.008**
Yes	11 (10.4)	11 (3.9)				
No	95 (89.6)	274 (96.1)				
Postoperative complications			1.39 (1.108–1.769)	0.016**	1.24 (1.123–1.439)	0.025*
Yes	46 (43.1)	88 (30.9)				
No	60 (56.9)	197 (69.1)				

*Note:* The Sar group refers to the group with sarcopenia before surgery. The N‐Sar group refers to the group without sarcopenia before surgery. Adjusted risk ratio: adjusted for MMSE, pre‐NCD, MNA, ADL, frailty and ASA classification.

Abbreviations: APCI, acute postoperative cognitive impairment; ICU, intensive care unit; RR, risk ratio.

^a^ Values are reported as *n* (%) unless otherwise indicated.

* *p* < 0.05.

** *p* < 0.01.

*** *p* < 0.001.

### 3.6 Higher Rate of Falls and Mortality in the Sarcopenia Group Within Six Months

Among the 391 patients who completed 6‐month follow‐up, patients in the sarcopenia group had a significantly higher risk of falls compared with those without sarcopenia (adjusted RR = 2.89; 95% CI: 1.546–5.078; *p* = 0.028). Similarly, 6‐month all‐cause mortality was substantially elevated in the sarcopenia group (adjusted RR = 3.07; 95% CI: 1.165–7.872; *p* = 0.016). No significant difference was observed in the rate of hospital readmission between groups (Tables 5 and S6).

**TABLE 5:** Unadjusted RR and adjusted RR values for postoperative 6 mouths outcomes of the sarcopenia group and not sarcopenia group.

Outcome of 6 months	Sar group *n* = 106 (%)^a^	N‐Sar group *n* = 285 (%)^a^	Unadjusted RR (95% CI)	*p*	Adjusted RR (95% CI)	*p*
Rehospitalization			0.91 (0.817–1.055)	0.651	0.98 (0.917–1.115)	0.674
Yes	12 (11.4)	36 (12.6)				
No	94 (88.6)	249 (87.4)				
Fall			3.02 (1.443–7.020)	0.036*	2.89 (1.546–5.078)	0.028*
Yes	15 (14.2)	13 (4.7)				
No	91 (85.8)	272 (95.3)				
Death			3.66 (1.219–8.355)	0.014*	3.07 (1.165–7.872)	0.016*
Yes	7 (6.6)	5 (1.8)				
No	99 (93.4)	280 (98.2)				

*Note:* The Sar group refers to the group with sarcopenia before surgery. The N‐Sar group refers to the group without sarcopenia before surgery. Adjusted risk ratio: adjusted for MMSE, pre‐NCD, MNA, ADL, frailty and ASA classification.

Abbreviations: CI, confidence interval; RR, risk ratio.

^a^ Values are reported as *n* (%) unless otherwise indicated.

* *p* < 0.05.

** *p* < 0.01.

*** *p* < 0.001.

### 3.7 Shorter Overall Survival Time in the Sarcopenia Group

**FIGURE 1:**
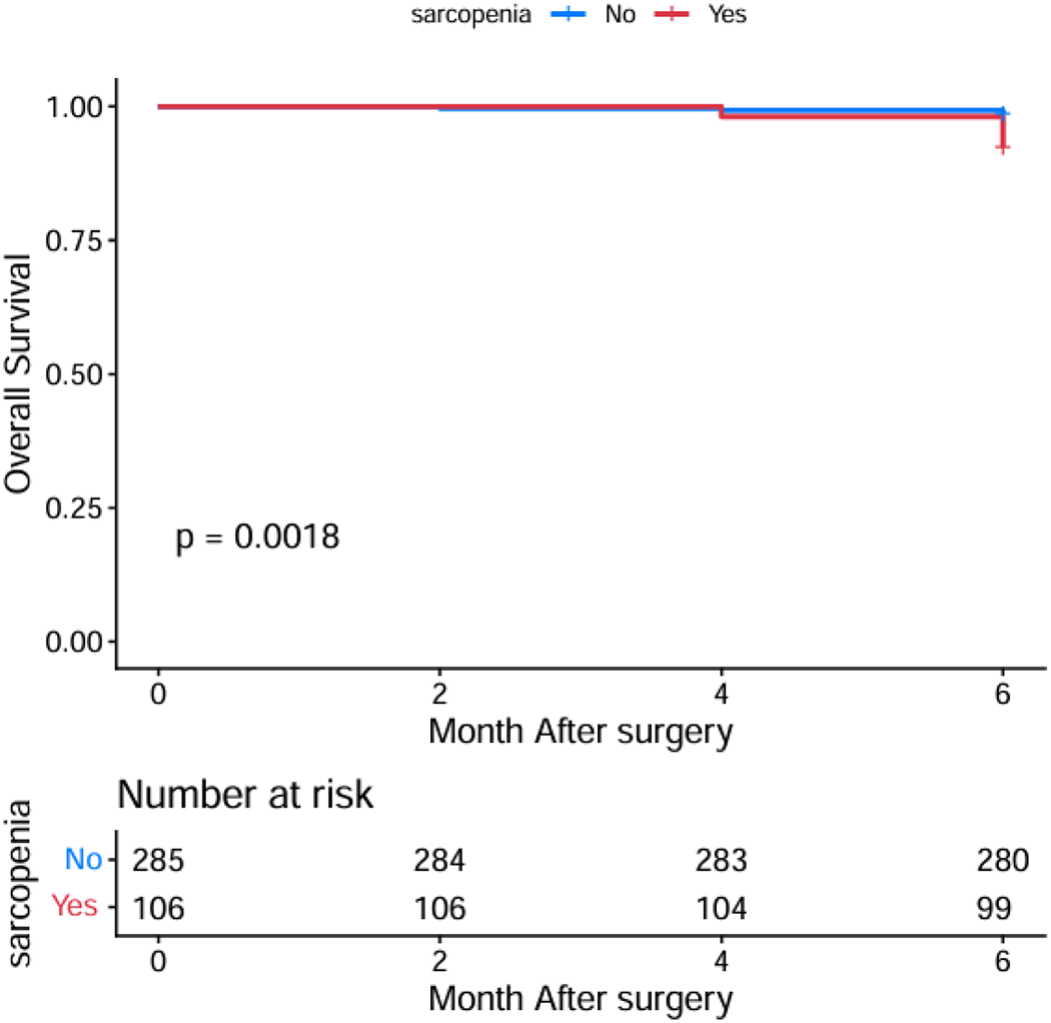
Length of overall survival time dependent on with or without sarcopenia.

To preliminarily assess the potential impact of sarcopenia on long‐term prognosis, we performed an exploratory Kaplan–Meier survival analysis. The endpoint event was all‐cause death within 6 months after surgery, and survival time was defined as the time (in months) from hospital discharge to death or the last follow‐up. Survival curves for the sarcopenia and non‐sarcopenia groups were plotted using the survival and survminer packages in R software (version 4.3.1), and the log5‐rank test was used to compare survival differences between the two groups. Compared with the non‐sarcopenia group, the sarcopenia group showed a decreased six‐month postoperative survival rate (*p* = 0.0018), as shown in Figure 1.

### 3.8 Exploratory Findings: Gut Microbial and Metabolic Signatures Associated With Sarcopenia‐Related Acute Cognitive Decline

In an exploratory sub‐analysis of 60 patients with available preoperative faecal samples, we compared gut microbial composition and metabolic profiles between those who developed acute postoperative cognitive decline (APCI group, *n* = 30) and those who did not (N‐APCI group, *n* = 30), with a further subgroup analysis focusing on patients with preoperative sarcopenia who developed cognitive decline (S‐APCI, *n* = 19) versus non‐sarcopenic controls without cognitive decline (NS‐NAPCI, *n* = 19).

### 3.9 Microbial Composition

**FIGURE 2:**
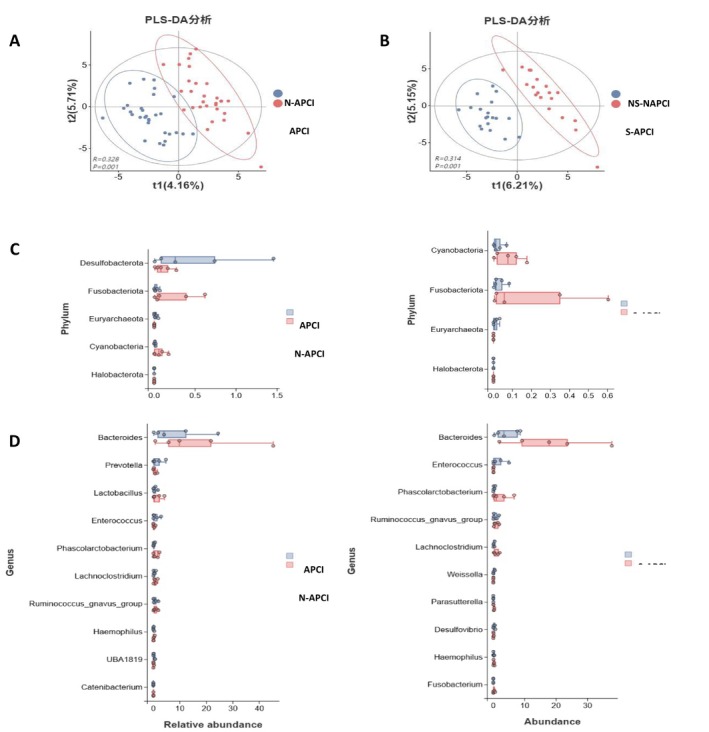
Differential species analysis of gut microbiota. (a) Beta diversity analysis between the APCI group and the N‐APCI group; (b) beta diversity analysis between the S‐APCI group and the NS‐NAPCI group. (c) Differential species at the phylum level; (d) differential species at the genus level (only the top 10 genera in terms of relative abundance are shown). The overall structural differences in gut microbiota between the two groups were compared using the PLS‐DA model, combined with Adonis analysis for inter‐group comparisons.

While alpha diversity did not differ significantly between groups, beta diversity analysis using PLS‐DA revealed distinct clustering of microbial communities in both the overall APCI versus N‐APCI comparison and the S‐APCI versus NS‐NAPCI subgroup comparison (Adonis test, *p* < 0.05; Figure 2A,B). At the phylum level, the Firmicutes/Bacteroidetes (F/B) ratio was elevated in both the APCI group and the S‐APCI subgroup. Specifically, the S‐APCI subgroup exhibited significantly reduced relative abundance of Fusobacteriota and Cyanobacteria compared with NS‐NAPCI controls (both *p* < 0.05; Figure 2C). At the genus level, the S‐APCI subgroup showed a marked decrease in the abundance of Bacteroides, Lachnoclostridium and Phascolarctobacterium, alongside increased abundance of Enterococcus and Weissella (all *p* < 0.05; Figure 2D, Tables S7–S9).

### 3.10 Metabolic Alterations

**FIGURE 3:**
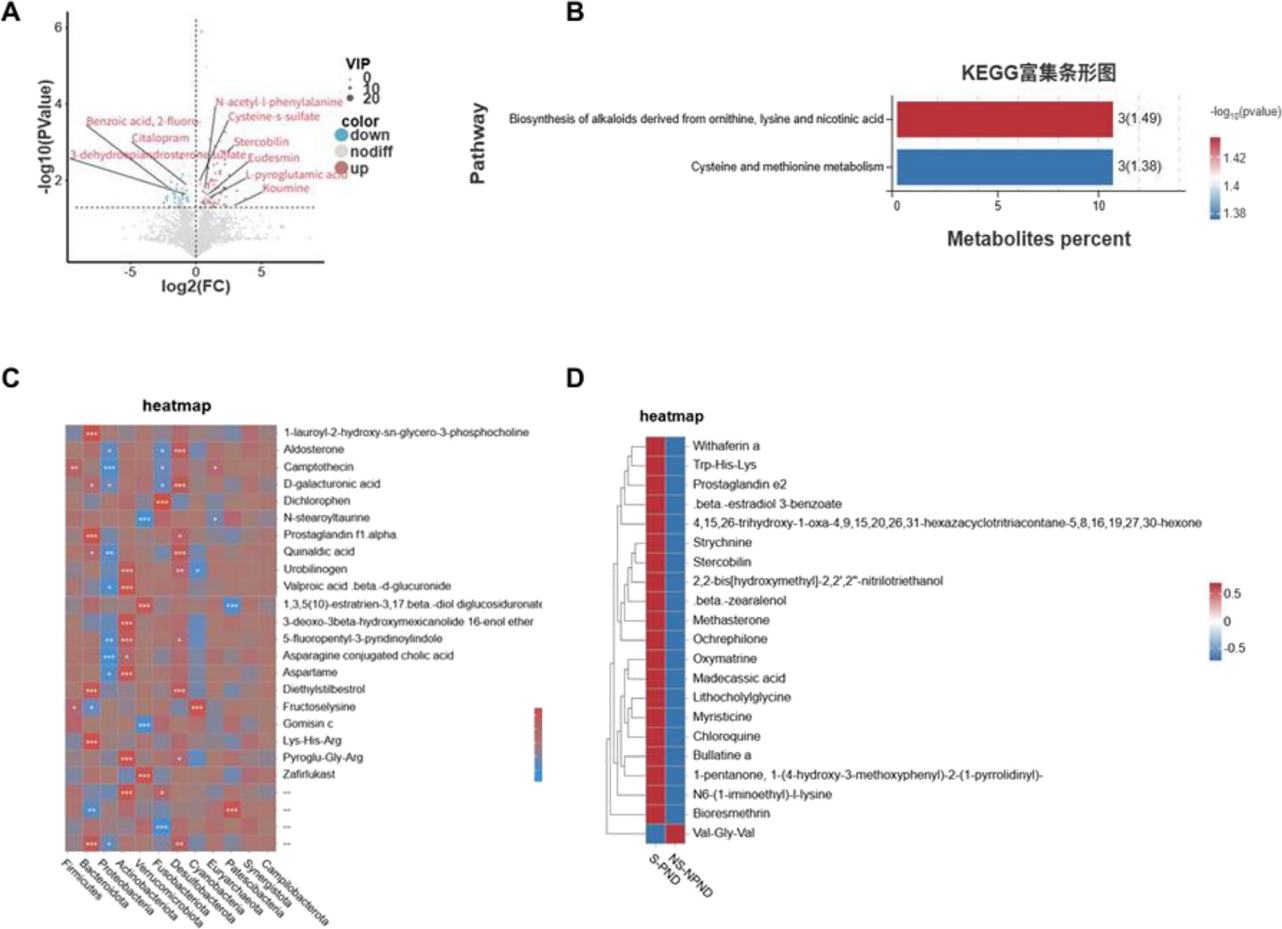
Differential metabolite analysis: (A) Volcano plot of APCI versus N‐APCI; (B) heatmap of differential metabolites between S‐APCI and NS‐NAPCI (top 20); (C) KEGG enrichment bubble plot; (D) microbiota–metabolite association heatmap (Spearman correlation).

Untargeted metabolomics revealed significant alterations in faecal metabolite profiles. In both the overall APCI group and the S‐APCI subgroup, stercobilin—a bilirubin catabolite—and *β*‐estradiol 3‐benzoate were consistently upregulated (*p* < 0.05; Figure 3A,B). KEGG pathway enrichment analysis in the S‐APCI subgroup suggested potential alterations in cysteine and methionine metabolism and biosynthesis of alkaloids derived from ornithine, lysine and nicotinic acid (*p* < 0.05; Figure 3C).

### 3.11 Microbe–Metabolite Associations

Spearman correlation analysis in the S‐APCI subgroup revealed that Fusobacteriota abundance was negatively correlated with aldosterone levels, whereas urobilinogen was positively correlated with Actinobacteriota and Desulfobacterota (*p* < 0.05; Figure 3D). These findings suggest that preoperative gut dysbiosis and altered microbial metabolite production may contribute to the heightened vulnerability to acute cognitive decline in sarcopenic patients.

## 4 Discussion

This study identified preoperative frailty and sarcopenia as independent risk factors for acute postoperative cognitive decline in older adults undergoing noncardiac surgery. Notably, the primary outcome defined in this study—a ≥ 2‐point decrease in MMSE on postoperative Day 3—captures an acute cognitive perturbation occurring in the immediate postoperative window. According to the 2018 consensus nomenclature, this temporal profile corresponds most directly to POD rather than the more prolonged entity of POCD. While we utilized the MMSE for standardized cognitive screening, we acknowledge that without formal Confusion Assessment Method (CAM) testing, we cannot distinguish between pure subsyndromal delirium and acute cognitive decline without attentional deficits. Nevertheless, in clinical practice, any significant acute drop in MMSE in an older surgical patient is highly suggestive of underlying delirium pathophysiology. Follow‐up observations revealed that patients with preoperative sarcopenia experienced worse postoperative outcomes, including a higher incidence of acute cognitive decline, increased ICU transfer rates, more postoperative complications and higher 6‐month mortality and fall rates.

The findings of this study are consistent with previous research indicating that sarcopenia serves as an independent risk factor for acute postoperative cognitive disturbances. For example, a cohort study evaluating the preoperative psoas muscle size in older surgical patients found that reduced muscle mass was significantly associated with the development of postoperative delirium [21]. Similarly, a retrospective cohort study investigating the link between preoperative low skeletal muscle mass (LSMM) and POD in older adult patients undergoing colorectal cancer surgery identified LSMM as a risk factor for POD [12]. The present study extends these observations by demonstrating that sarcopenia, defined according to AWGS criteria and assessed via whole‐body bioelectrical impedance analysis, similarly predicts early postoperative cognitive decline in a broader noncardiac surgical population.

The elevated incidence of acute postoperative cognitive decline in sarcopenic older adults may be attributed to multiple interrelated mechanisms. Briefly, frailty and sarcopenia synergistically impair physiological reserve and stress adaptation [22, 23, 24], chronic low‐grade inflammation, characterized by elevated IL‐6, IL‐8 and TNF‐α, primes the central nervous system for an exaggerated neuroinflammatory response to surgical insult; malnutrition and immune dysfunction further compromise neurotrophic support and wound healing; and reduced cardiopulmonary reserve increases intraoperative susceptibility to cerebral hypoperfusion [13, 25, 26]. Beyond these established pathways, our exploratory multi‐omics findings provide novel insights into the potential role of the gut–muscle–brain axis.

In the subset of patients who underwent preoperative faecal sampling, sarcopenic individuals who developed acute cognitive decline exhibited a distinct gut microbial signature, including an elevated Firmicutes/Bacteroidetes (F/B) ratio and a marked reduction in Bacteroides abundance. An increased F/B ratio is a recognized indicator of gut dysbiosis and has been linked to impaired intestinal barrier function and systemic inflammation [27]. Bacteroides species are major producers of short‐chain fatty acids (SCFAs), particularly butyrate, which exerts anti‐inflammatory effects and supports blood–brain barrier integrity. Depletion of Bacteroides may therefore diminish SCFA‐mediated neuroprotection, rendering the aging brain more vulnerable to surgical stress [28].

Notably, we also observed a significant upregulation of faecal stercobilin—a terminal catabolite of heme metabolism—in the S‐APCI subgroup. Stercobilin is generated through the sequential action of heme oxygenase, biliverdin reductase and gut microbial bilirubin reductases. Elevated faecal stercobilin may reflect either increased heme turnover or altered microbial bilirubin metabolism. Unconjugated bilirubin can cross the blood–brain barrier and, at supraphysiological concentrations, exert neurotoxic effects via oxidative stress and mitochondrial dysfunction [29]. Furthermore, Firmicutes‐encoded BilR enzymes are key mediators of bilirubin reduction; the elevated F/B ratio in our cohort may thus directly contribute to dysregulated bilirubin catabolism with potential neurocognitive consequences [30].

Additionally, we detected increased levels of *β*‐estradiol 3‐benzoate and other oestrogen derivatives in the S‐APCI subgroup. Elevated faecal excretion of conjugated oestrogens often signifies impaired enterohepatic recirculation and reduced systemic bioavailability—a phenomenon linked to gut dysbiosis. In sarcopenic patients, who already exhibit declining sex hormone levels, diminished systemic oestrogen availability could further compromise synaptic plasticity and neurotrophic support.

Collectively, these exploratory findings suggest that preoperative sarcopenia may prime the gut–muscle–brain axis towards a pro‐inflammatory, metabolically dysregulated state that lowers the threshold for acute cognitive decompensation following surgical stress. However, given the cross‐sectional nature of our microbiome sampling and the modest sample size, causal inferences cannot be drawn. Based on recent studies, we further speculate that sarcopenia and frailty may trigger the upregulation of the ANXA1‐FPR2 signalling pathway, promoting the expansion and spatial redistribution of myeloid‐derived suppressor cells (MDSCs). This in turn exacerbates systemic chronic inflammation, intestinal barrier impairment and neuroinflammation, amplifies central nervous system damage caused by surgical stress and ultimately increases the risk of postoperative cognitive decline [31]. Future studies incorporating longitudinal faecal sampling and experimental models are essential to validate these associations. The incidence of postoperative complications during hospitalization was significantly higher in the sarcopenia group. This finding aligns with prior research, including a study of 234 patients undergoing liver transplantation for malignant tumours, which found that hospitalization morbidity was significantly higher in sarcopenia patients [32]. Possible explanations include the following: (1) reduced amino acid production and release from skeletal muscles in sarcopenic patients, leading to insufficient raw materials for liver protein synthesis and decreased glutamine supply, which is essential for immune cell activity; (2) a reduced ability to eliminate free radicals in sarcopenic patients, resulting in exacerbated tissue damage post‐surgery if excessive free radicals are not adequately cleared [33]; (3) Lute et al. suggest that skeletal muscle atrophy can lead to decreased anti‐inflammatory factors such as IL‐15, while increasing adipokines and inflammatory factors inhibit NK cells and impair immune function, raising the risk of perioperative infections, poor wound healing, and delayed recovery [34]. The duration of surgery, postoperative oral intake time, type of surgery and demographic data may all have a certain impact on the patient's muscle mass to a certain extent. Other surgery‐related factors may also influence the progression of sarcopenia. Therefore, patients with sarcopenia may experience a worsened state after surgery, exacerbating the severity of the disease and potentially correlating with adverse postoperative outcomes, although this has not been definitively proven.

Six‐month follow‐up data revealed a significantly higher incidence of falls among patients in the sarcopenia group. This observation is consistent with a prior study of 1110 community‐dwelling older adults, which reported a robust positive association between sarcopenia and falls risk [35]. The heightened fall susceptibility in sarcopenic individuals is multifactorial, primarily driven by diminished lower limb muscle strength, impaired postural reflexes and compromised balance control. Notably, the consequences of falls in this population can be devastating, often precipitating a cascade of immobilization, fracture and accelerated functional decline. Given these risks, early postoperative rehabilitation strategies—including resistance training and balance exercises—should be prioritized for sarcopenic patients to mitigate fall‐related morbidity and preserve long‐term independence. Our study incorporated six secondary outcomes to comprehensively evaluate the perioperative and longer‐term impact of sarcopenia. While 6‐month readmission, falls and mortality are established adverse correlates of sarcopenia, the present study offers unique value by delineating a putative causal chain: preoperative sarcopenia → acute postoperative cognitive decline → increased 6‐month falls and mortality. This framework identifies acute cognitive deterioration as a critical intermediate link between baseline muscle health and long‐term functional decline. Furthermore, falls within 6 months of discharge represent a sensitive, patient‐centred endpoint that directly captures the synergistic impairment of brain–muscle crosstalk. Unlike mortality, falls are potentially modifiable through targeted postoperative rehabilitation, rendering our findings clinically actionable for improving quality of life in this vulnerable population.

Previous studies examining the relationship between sarcopenia and postoperative cognition have largely been cross‐sectional, retrospective or limited to animal models. The present study offers several methodological strengths. First, we employed a prospective cohort design, which minimizes recall bias and establishes temporal precedence between preoperative sarcopenia and subsequent outcomes. Second, unlike prior investigations that relied on CT‐derived measurements of localized muscle compartments (e.g., psoas area), we diagnosed sarcopenia according to the Asian Working Group for Sarcopenia criteria, utilizing whole‐body bioelectrical impedance analysis to quantify appendicular skeletal muscle mass. Third, our study extended follow‐up beyond in‐hospital events to capture patient‐centred functional outcomes, specifically post‐discharge falls—a clinically meaningful endpoint that is often overlooked in surgical cohort studies yet directly reflects the real‐world consequences of impaired brain–muscle crosstalk.

This study also has several limitations that warrant consideration. First, as an observational study, residual confounding cannot be excluded despite multivariate adjustment. Second, cognitive function was assessed only at postoperative Day 3, which captures acute cognitive perturbation but precludes evaluation of long‐term POCD. Extended cognitive follow‐up is needed to determine whether preoperative sarcopenia independently influences longer‐term cognitive trajectories. Third, patients with postoperative hospital stays shorter than 3 days could not undergo the scheduled cognitive assessment, potentially introducing selection bias. Fourth, the gut microbiome and metabolomics sub‐study was exploratory in nature, conducted on a small subset of patients (*n* = 60) and lacking postoperative faecal sampling; therefore, causal inferences regarding the gut–muscle–brain axis cannot be drawn, and these findings should be interpreted as hypothesis‐generating rather than confirmatory.

Despite these limitations, this study provides robust evidence that preoperative sarcopenia independently predicts acute postoperative cognitive decline and poor functional outcomes in older surgical patients, underscoring the value of sarcopenia screening in preoperative risk stratification.

## 5 Conclusions

This prospective cohort study demonstrates that preoperative sarcopenia is an independent risk factor for acute postoperative cognitive impairment—an early manifestation within the spectrum of perioperative neurocognitive disorders—in older adult surgical patients, alongside frailty. Patients with sarcopenia exhibited significantly higher rates of early cognitive impairment, postoperative complications, ICU admissions and 6‐month mortality compared with their non‐sarcopenia counterparts. Notably, sarcopenia was also associated with an increased incidence of falls during the 6‐month follow‐up period, underscoring its broader implications for postoperative recovery and long‐term functional decline.

These findings highlight the critical need for preoperative sarcopenia screening in geriatric surgical populations to identify high‐risk patients and implement targeted interventions. Future multicentre studies with larger cohorts and standardized neurocognitive assessments—including long‐term follow‐up to evaluate POCD—are warranted to validate these associations and elucidate underlying mechanisms. Additionally, exploration of sarcopenia‐specific therapies, such as resistance training, nutritional optimization and pharmacological strategies, may mitigate perioperative neurocognitive vulnerability and improve surgical outcomes in this high‐risk population.

## Funding

This study was supported by the Foundation of Chengdu Medical College (24LHLNYX1‐13, 24LHLNYX1‐24), Chengdu Key R&D Support Program for Technological Innovation (2024‐YF05‐00911‐SN); Sichuan Medical health Promotion Association general project (KY2022QN0289) and Chengdu Medical Research Projects (2023540).

## Disclosure

All authors have read and approved this version of the article, and due care has been taken to ensure the integrity of the work. Neither the entire paper nor any part of its content has been published or has been accepted elsewhere. It is not being submitted to any other journal.

## Ethics Statement

All of the participants signed an informed consent form before data or sample collection, according to the Declaration of Helsinki. The conduction of CSAC and this specific study was approved by the ethics committee of the First Affiliated Hospital of Chengdu Medical College (approval number 2021CYFYIRB‐BA‐67‐01).

## Conflicts of Interest

The authors declare no conflicts of interest.

## Supporting information


**Table S1:** Intraoperative variables of the Acute postoperative cognitive impairment group and Not Acute postoperative cognitive impairment group.
**Table S2:** Analysis of postoperative outcomes of the APCI and N‐APCI group.
**Table S3:** Univariate regression analysis for risk factors of APCI.
**Table S4:** Operative details of the Sar group and N‐Sar group.
**Table S5:** Postoperative short term outcomes of the Sar group and N‐Sar group.
**Table S6:** Postoperative 6 mouths outcomes of the Sar group and N‐Sar group.
**Table S7:** Complete differential abundance of gut microbiota at the phylum level between groups.
**Table S8:** Complete differential abundance of gut microbial at the genus level between the APCI and N‐APCI groups.
**Table S9:** Complete differential abundance of gut microbial at the genus level between the S‐APCI and NS‐NAPCI groups.
**Table S10:** Differential faecal metabolites between the APCI and N‐APCI groups (*p* < 0.05).
**Table S11:** Differential faecal metabolites between the S‐APCI and NS‐NAPCI groups (*p* < 0.05).
**Table S12:** KEGG pathway enrichment analysis of differential metabolites between the APCI and N‐APCI groups.

**Figure S1:** Paired MMSE Scores Preoperation and Postoperation (*n* = 443).The plot shows paired preoperative (Pre‐MMSE) and postoperative (Post‐MMSE) Mini‐Mental State Examination (MMSE) scores for 443 patients. Blue points represent preoperative MMSE scores, while red points represent postoperative MMSE scores. Black lines indicate cases where postoperative scores decreased compared with preoperative scores, while grey lines indicate cases where postoperative scores remained the same or increased. The size of each point reflects the repeat count, indicating the number of occurrences of specific MMSE scores across patients.
**Figure S2:** Flow chart of the study.
