# Supplementary material for: Sarcopenia Predicts Postoperative Cognitive Impairment and Poor Surgical Outcomes in Older Adults: A Prospective Cohort Study
Source: J Cachexia Sarcopenia Muscle. 2026 Jul 15;17(4):e70346. doi: 10.1002/jcsm.70346 (PMC13372572; doi:10.1002/jcsm.70346)
Supplement: Supplementary file 1 — Table S1: Intraoperative variables of the Acute postoperative cognitive impairment group and Not Acute postoperative cognitive impairment group. Table S2: Analysis of postoperative outcomes of the APCI and N‐APCI group. Table S3: Univariate regression analysis for risk factors of APCI. Table S4: Operative details of the Sar group and N‐Sar group. Table S5: Postoperative short term outcomes of the Sar group and N‐Sar group. Table S6: Postoperative 6 mouths outcomes of the Sar group and N‐Sar group. Table S7: Complete differential abundance of gut microbiota at the phylum level between groups. Table S8: Complete differential abundance of gut microbial at the genus level between the APCI and N‐APCI groups. Table S9: Complete differential abundance of gut microbial at the genus level between the S‐APCI and NS‐NAPCI groups. Table S10: Differential faecal metabolites between the APCI and N‐APCI groups (p < 0.05). Table S11: Differential faecal metabolites between the S‐APCI and NS‐NAPCI groups (p < 0.05). Table S12: KEGG pathway enrichment analysis of differential metabolites between the APCI and N‐APCI groups. [file JCSM-17-e70346-s001.docx]

Supplementary Table 1 Intraoperative variables of the **Acute postoperative cognitive impairment group and Not Acute postoperative cognitive impairment group**

| Intraoperative General Information | Total cohort n=443（%）^a^ | APCI^1^ group n=121（%）^a^ | N-APCI^2^ group n=322（%）^a^ | *P* |
| --- | --- | --- | --- | --- |
| Anesthesia Method |  |  |  | 0. 076 |
| General anesthesia | 336（75. 8） | 86（71. 1） | 250（77. 6） |  |
| Intraspinal anesthesia | 103（23. 3） | 32（26. 4） | 71（22. 0） |  |
| Nerve block | 4（0. 9） | 3（2. 5） | 1（0. 3） |  |
| ASA |  |  |  | 0. 261 |
| Ⅱ | 252（56. 9） | 69（57. 0） | 183（56. 8） |  |
| Ⅲ | 191（43. 1） | 52（43. 0） | 139（43. 2) |  |
| Surgical Classification |  |  |  | 0. 164 |
| Otorhinolaryngology | 15（3. 4） | 6（5. 0） | 9（2. 8） |  |
| Gynecology | 29（5. 5） | 4（3. 3） | 25（7. 8） |  |
| General Surgery | 199（44. 9） | 59（48. 8） | 140（43. 5） |  |
| Orthopedics | 25（5. 8） | 6（5. 0） | 19 （5. 9) |  |
| Urology | 110（24. 8） | 34（28. 1） | 76（23. 6） |  |
| Burn and Plastic Surgery | 8（1. 8） | 3（2. 5） | 5（1. 6） |  |
| Thoracic and Cardiac Surgery | 53（12. 0） | 8（6. 6） | 45（14. 0） |  |
| Others | 4（0. 9） | 1（0. 8） | 3（0. 9） |  |
| Dexmedetomidine |  |  |  | 0. 776 |
| Yes | 140（30. 3） | 37（30. 6） | 103（32. 0） |  |
| No | 303（69. 7） | 84（69. 4） | 219（68. 0） |  |
| Analgesic Pump |  |  |  | 0. 401 |
| Yes | 245（55. 3） | 63 （52. 1） | 182（56. 5） |  |
| No | 198（44. 7） | 58 （47. 9） | 140（43. 5） |  |
|  |  |  |  |  |
| Nerve Block |  |  |  | 0. 742 |
| Yes | 196（44. 2） | 52（43. 0） | 144 （44. 7） |  |
| No | 247（55. 8） | 69 （57. 0%） | 178 （55. 3） |  |
| Blood Loss, ml | 20（10-50） | 20（10-50） | 20（10-50） | 0. 860 |
| Anesthesia Duration，min^b^ | 150（100-257） | 150（102-234） | 150（100-267） | 0. 466 |
| Operation Duration，min | 108（65-200） | 106（65-180） | 110（63-210) | 0. 582 |
| Fluid Replacement，ml | 1100（1000-2100） | 1100（1000-2000) | 1200（1000-2100） | 0. 411 |

**Note:** Abbreviations: **APCI: Acute postoperative cognitive impairment;** ASA: American Society of Anesthesiologists.

1The APCI group refers to the group with **acute postoperative cognitive impairmen**t; 2 N-APCI group refers to the group without **acute postoperative cognitive impairment**.

^a^Values are reported as No.(%)unless otherwise indicated.

^b^Defined as the interval from administration of anesthetic to pull out tracheal tube (Defined as the interval from administration of anesthetic to discharge from operating room).

Supplementary Table 2 Analysis of postoperative outcomes of the **APCI** and N-**APCI** group

| Postoperative Status | Total cohort n=443(%)^a^ | **APCI**^1^ group n=121(%)^a^ | N-**APCI**^2^ group n=322(%)^a^ | *P* |
| --- | --- | --- | --- | --- |
| Transferred to ICU |  |  |  | 0. 316 |
| Yes | 25（5. 6） | 9（7. 4） | 16（5. 0） |  |
| No | 418（94. 4） | 112（92. 6） | 306（95. 0） |  |
| Postoperative Complications |  |  |  | 0. 218 |
| Yes | 152（41. 3） | 47（38. 8） | 105（32. 6） |  |
| No | 291（58. 7） | 74（61. 2） | 217（67. 4） |  |
| Length of Hospital Stay, days | 12. 44±6. 12 | 12. 02±5. 56 | 12. 93±7. 27 | 0. 213 |

**Note:** Abbreviations: **APCI: Acute postoperative cognitive impairment;** ICU: Intensive care unit.

1 The **APCI** group refers to the group with **acute postoperative cognitive impairment**; 2. N-**APCI** group refers to the group without **acute postoperative cognitive impairment.**

^a^Values are reported as No.(%)unless otherwise indicated.

Supplementary Table 3 Univariate regression analysis for risk factors of **APCI**

| Variables | OR^1^ | 95% CI^2^ | P |
| --- | --- | --- | --- |
| Age | 1. 031 | 0. 996-1. 067 | 0. 088 |
| Weight | 1. 012 | 0. 988-1. 036 | 0. 339 |
| BMI | 1. 033 | 0. 960-1. 112 | 0. 388 |
| Gender | 0. 780 | 0. 460-1. 325 | 0. 359 |
| Education Level | 1. 075 | 0. 833-1. 387 | 0. 580 |
| Pre-NCD | 1. 471 | 0. 946-1. 902 | 0. 023^*^ |
| Frailty | 5. 427 | 1. 699-7. 342 | 0. 004^**^ |
| Degree of Frailty | 0. 581 | 0. 249-1. 360 | 0. 211 |
| Hypertension | 1. 648 | 0. 975-2. 788 | 0. 062 |
| Diabetes | 0. 888 | 0. 488-1. 616 | 0. 697 |
| Sarcopenia | 3. 939 | 2. 505-6. 194 | ＜0. 001^***^ |
| Anesthesia Method | 0. 564 | 0. 308-1. 033 | 0. 064 |
| ASA | 0. 961 | 0. 558-1. 654 | 0. 886 |
| Classification of Surgical Procedures | 0. 940 | 0. 800-1. 105 | 0. 452 |
| Dexmedetomidine | 0. 617 | 0. 349-1. 091 | 0. 097 |
| Analgesic Pump | 0. 932 | 0. 611-1. 420 | 0. 742 |
| Nerve Block | 0. 836 | 0. 549-1. 271 | 0. 401 |
| Blood Loss | 1. 000 | 0. 999-1. 001 | 0. 690 |
| Anesthesia Duration | 0. 995 | 0. 987-1. 003 | 0. 243 |
| Operation Duration | 1. 005 | 0. 996-1. 014 | 0. 293 |
| ICU | 0. 946 | 0. 249-3. 588 | 0. 935 |
| Postoperative Complications | 1. 281 | 0. 740-2. 218 | 0. 376 |
| Total Length of Hospital Stay | 0. 984 | 0. 983-1. 033 | 0. 519 |

**Note:** Abbreviations: **APCI: Acute postoperative cognitive impairment**; BMI: Body mass index; MMSE: Mini-Mental State Examination; Pre-NCD: Pre-existing neurocognitive disorders; ASA: American Society of Anesthesiologists;ICU: Intensive care unit.

1 OR: Odds ratio; 2 CI: Confidence interval.

^*^*P*<0.05，^**^*P*<0.01，^***^*P*<0.001.

Supplementary Table 4 Operative details of the Sar group and N-Sar group

| Intraoperative Conditions | Total cohort  n=391(%)^a^ | Sar group^1^  n=106(%)^a^ | N-Sar group^2^  n=285(%)^a^ | *P* |
| --- | --- | --- | --- | --- |
| Type of Surgery |  |  |  | 0. 642 |
| Otolaryngology | 12（3. 7） | 4（3. 8） | 8（2. 8） |  |
| Gynecology | 26（66. 5） | 6（5. 7） | 20（6. 9） |  |
| General Surgery | 176（45. 0） | 49（46. 2） | 127（44. 6） |  |
| Orthopedics | 22（56. 3） | 5（4. 7） | 17（6. 0） |  |
| Urology | 97（24. 8） | 31（29. 3） | 66（23. 2） |  |
| Burn and Plastic Surgery | 7（1. 8） | 2（1. 9） | 5（1. 8） |  |
| Thoracic Surgery | 47（12. 0） | 8（7. 6） | 39（13. 7） |  |
| Others | 4（1. 0） | 1（0. 9） | 3（1. 0） |  |
| Type of Anesthesia |  |  |  | 0. 560 |
| Neural Blockade | 4（1. 0） | 2（1. 9） | 2（0. 6） |  |
| Intraspinal Anesthesia | 91（23. 3） | 26（24. 5） | 65（22. 8） |  |
| General Anesthesia | 296（75. 7） | 78（73. 6） | 218（76. 6） |  |
| ASA |  |  |  | 0. 043^*^ |
| Ⅱ | 222（56. 8） | 53（50. 0） | 169（59. 3） |  |
| Ⅲ | 169（43. 2） | 53（50. 0） | 116（40. 7） |  |
| Dexmedetomidine |  |  |  | 0. 162 |
| Yes | 124（31. 2） | 39（36. 8） | 85（29. 8） |  |
| No | 267（68. 8） | 67（63. 2） | 200（70. 2） |  |
| Neural Blockade |  |  |  | 0. 736 |
| Yes | 173（44. 3） | 48（45. 3） | 125（43. 9） |  |
| No | 218（55. 7） | 58（54. 7） | 160（56. 1） |  |
| Use of Analgesia Pump |  |  |  | 0. 134 |
| Yes | 216（55. 2） | 52（49. 1） | 164（57. 5） |  |
| No | 175（44. 8） | 54（50. 9） | 121（42. 5） |  |
| Median Operation Time (IQR), min | 110（63-203） | 120（60-210） | 107（63-203） | 0. 708 |
| Median Anesthesia Time (IQR), min^b^ | 15（100-260） | 165（100-260） | 150（100-260） | 0. 937 |
| Median Blood Loss (IQR), ml | 20（10-50） | 20（10-50） | 20（10-50） | 0. 530 |
| Median Fluid Replacement Volume (IQR), ml | 1200（1000-2100） | 1100（1000-2100） | 1200（1000-2100） | 0. 561 |

**Note:** Abbreviations: ASA: American Society of Anesthesiologists.

1 The Sar group refers to the group with sarcopenia before surgery; 2 The N-Sar group refers to the group without sarcopenia before surgery.

^a^Values are reported as No.(%)unless otherwise indicated.

^b^Defined as the interval from administration of anesthetic to pull out tracheal tube (Defined as the interval from administration of anesthetic to discharge from operating room).

^*^*P*<0.05，^**^*P*<0.01，^***^*P*<0.001.

Supplementary Table 5 Postoperative short term outcomes of the Sar group and N-Sar group

| Postoperative Outcome | Total cohort  n=391(%)^a^ | Sar group^1^  n=106(%)^a^ | N-Sar group^2^  n=285(%)^a^ | *P* |
| --- | --- | --- | --- | --- |
| **APCI** |  |  |  | ＜0. 001^***^ |
| Yes | 119（30. 4） | 49（46. 3） | 70（24. 6） |  |
| No | 272（69. 6） | 57（53. 7） | 215（75. 4） |  |
| Transferred to ICU |  |  |  | 0. 005^**^ |
| Yes | 22（5. 6） | 11（10. 4） | 11（3. 9） |  |
| No | 369（94. 4） | 95（89. 6） | 274（96. 1） |  |
| Length of Hospital Stay | 12. 69±6. 85 | 12. 55±6. 22 | 12. 74±7. 09 | 0. 800 |
| Postoperative Complications |  |  |  | 0. 016^**^ |
| Yes | 134（34. 7） | 46（43. 1） | 88(30. 9) |  |
| No | 257（65. 3） | 60（56. 9） | 197(69. 1) |  |

**Note:** Abbreviations: **APCI: acute postoperative cognitive impairment**; ICU: Intensive care unit.

1 The Sar group refers to the group with sarcopenia before surgery; 2 The N-Sar group refers to the group without sarcopenia before surgery.

^a^Values are reported as No.(%)unless otherwise indicated.

^*^*P*<0.05，^**^*P*<0.01，^***^*P*<0.001.

Supplementary Table 6 Postoperative 6 mouths outcomes of the Sar group and N-Sar group

| Outcome of 6 months | Total cohort  n=391（%）^a^ | Sar group^1^  n=106（%）^a^ | N-Sar group^2^  n=285（%）^a^ | *P* |
| --- | --- | --- | --- | --- |
| Rehospitalization |  |  |  | 0. 651 |
| Yes | 48（12. 3） | 12（11. 4） | 36（12. 6） |  |
| No | 343（87. 7） | 96（88. 6） | 247（87. 4） |  |
| Fall |  |  |  | 0. 036^*^ |
| Yes | 28（7. 2） | 15（14. 2） | 13（4. 6） |  |
| No | 363（92. 8） | 91（85. 8） | 272（95. 4） |  |
| Death |  |  |  | 0. 014^*^ |
| Yes | 12（3. 1） | 7（6. 6） | 5（1. 8） |  |
| No | 379（96. 9） | 99（93. 4） | 280（98. 2） |  |

**Note:** Abbreviations: RR: Relative risk.

1The Sar group refers to the group with sarcopenia before surgery; 2The N-Sar group refers to the group without sarcopenia before surgery

^a^Values are reported as No. (%) unless otherwise indicated.

^*^*P*<0.05，^**^*P*<0.01，^***^*P*<0.001.

| Supplementary Table 7. Complete differential abundance of gut microbiota at the phylum level between groups. | | | | | |
| --- | --- | --- | --- | --- | --- |
| Phylum | N-APCI | APCI | NS-NAPCI | S-APCI | Domain |
| Firmicutes | 51.61 | 53.11 | 52.28 | 57.91 | Bacteria |
| Bacteroidota | 22.94 | 20.71 | 25.93 | 15.58 | Bacteria |
| Proteobacteria | 18.03 | 16.60 | 16.96 | 15.15 | Bacteria |
| Actinobacteriota | 3.66 | 6.59 | 1.85 | 9.01 | Bacteria |
| Verrucomicrobiota | 1.89 | 0.82 | 1.10 | 0.38 | Bacteria |
| Desulfobacterota | 0.37 | 0.73 | 0.53 | 0.61 | Bacteria |
| Fusobacteriota | 0.81 | 0.03 | 0.54 | 0.03 | Bacteria |
| Euryarchaeota | 0.00 | 0.66 | 0.00 | 0.42 | Archaea |
| Cyanobacteria | 0.27 | 0.28 | 0.38 | 0.44 | Bacteria |
| Patescibacteria | 0.06 | 0.03 | 0.06 | 0.04 | Bacteria |
| Other | 0.04 | 0.06 | 0.04 | 0.07 |  |
| Unclassified | 0.32 | 0.36 | 0.34 | 0.36 |  |

| Supplementary Table 8. Complete differential abundance of gut microbial at the genus level between the APCI and N-APCI groups. | | | | | | |
| --- | --- | --- | --- | --- | --- | --- |
| Genus | APCI | N-APCI | Fold  (N-APCI/APCI) | | p-value | q-value |
| Bacteroides | 9.480 | 15.329 | 1.617 | | 0.009 | 0.044 |
| Prevotella | 4.086 | 1.975 | 0.483 | | 0.035 | 0.112 |
| Lactobacillus | 2.842 | 2.486 | 0.875 | | 0.048 | 0.146 |
| Enterococcus | 3.854 | 0.576 | 0.149 | | 0.002 | 0.016 |
| Phascolarctobacterium | 0.796 | 2.059 | 2.586 | | 0.006 | 0.034 |
| Lachnoclostridium | 0.533 | 2.051 | 3.846 | | 0.001 | 0.012 |
| Ruminococcus_gnavus_group | 0.614 | 1.578 | 2.569 | | 0.008 | 0.040 |
| Haemophilus | 0.222 | 1.397 | 6.287 | | 0.005 | 0.033 |
| UBA1819 | 1.377 | 0.145 | 0.105 | | 0.022 | 0.077 |
| Catenibacterium | 0.044 | 1.145 | 25.822 | | 0.000 | 0.000 |
| Weissella | 0.843 | 0.085 | 0.101 | | 0.002 | 0.016 |
| Fusobacterium | 0.025 | 0.809 | 32.775 | | 0.000 | 0.001 |
| Desulfovibrio | 0.667 | 0.090 | 0.135 | | 0.000 | 0.005 |
| Barnesiella | 0.408 | 0.185 | 0.454 | | 0.020 | 0.076 |
| Parasutterella | 0.149 | 0.412 | 2.767 | | 0.000 | 0.007 |
| Methanobrevibacter | 0.452 | 0.001 | 0.003 | | 0.000 | 0.000 |
| Lachnospira | 0.335 | 0.098 | 0.293 | | 0.004 | 0.032 |
| Ruminococcus_gauvreauii_group | 0.103 | 0.267 | 2.603 | | 0.014 | 0.058 |
| Peptostreptococcus | 0.025 | 0.309 | 12.223 | | 0.003 | 0.021 |
| Pediococcus | 0.019 | 0.274 | 14.141 | | 0.000 | 0.000 |
| Eubacterium_ruminantium_group | 0.202 | 0.076 | 0.379 | | 0.007 | 0.039 |
| Methanosphaera | 0.211 | 0.000 | 0.002 | | 0.000 | 0.001 |
| UCG-005 | 0.133 | 0.067 | 0.508 | | 0.021 | 0.077 |
| Prevotellaceae_NK3B31_group | 0.002 | 0.182 | 117.358 | | 0.000 | 0.000 |
| Rikenellaceae_RC9_gut_group | 0.169 | 0.002 | 0.010 | | 0.000 | 0.000 |
| Faecalitalea | 0.012 | 0.144 | 12.107 | | 0.008 | 0.040 |
| Eisenbergiella | 0.027 | 0.127 | 4.760 | | 0.038 | 0.119 |
| Intestinimonas | 0.144 | 0.005 | 0.035 | | 0.001 | 0.012 |
| Aggregatibacter | 0.013 | 0.128 | 9.609 | | 0.000 | 0.000 |
| Lachnospiraceae_UCG-004 | 0.077 | 0.050 | 0.653 | | 0.002 | 0.016 |
| Allisonella | 0.017 | 0.059 | 3.469 | | 0.030 | 0.097 |
| Eubacterium_brachy_group | 0.057 | 0.019 | 0.329 | 0.020 | | 0.076 |
| Acidaminococcus | 0.032 | 0.035 | 1.111 | 0.006 | | 0.037 |
| Comamonas | 0.038 | 0.002 | 0.043 | 0.005 | | 0.034 |
| TM7x | 0.010 | 0.030 | 3.144 | 0.003 | | 0.021 |
| DTU089 | 0.015 | 0.013 | 0.893 | 0.014 | | 0.058 |
| Scardovia | 0.024 | 0.002 | 0.068 | 0.013 | | 0.056 |
| Catabacter | 0.006 | 0.014 | 2.248 | 0.027 | | 0.089 |
| Lachnospiraceae_ND3007_group | 0.009 | 0.010 | 1.136 | 0.010 | | 0.048 |
| Myroides | 0.018 | 0.000 | 0.000 | 0.022 | | 0.077 |
| Anaeroglobus | 0.002 | 0.014 | 6.898 | 0.012 | | 0.051 |
| Actinidia_chinensis | 0.000 | 0.013 | ＜0.001 | 0.001 | | 0.014 |
| Herbinix | 0.011 | 0.000 | 0.028 | 0.000 | | 0.003 |
| Methanocorpusculum | 0.010 | 0.000 | 0.000 | 0.011 | | 0.050 |
| Dysgonomonas | 0.007 | 0.001 | 0.109 | 0.017 | | 0.066 |
| Epulopiscium | 0.007 | 0.000 | 0.014 | 0.011 | | 0.050 |
| Gram-negative_bacterium_cTPY-13 | 0.000 | 0.007 | ＜0.001 | 0.006 | | 0.034 |
| Cellulosilyticum | 0.006 | 0.000 | 0.008 | 0.023 | | 0.077 |

| Supplementary Table 9. Complete differential abundance of gut microbial at the genus level between the S-APCI and NS-NAPCI groups. | | | | | |
| --- | --- | --- | --- | --- | --- |
| Genus | S-APCI | NS-NAPCI | Fold  (NS-NAPCI/  S-APCI) | p-value | q-value |
| Bacteroides | 7.059 | 18.787 | 2.661 | 0.000 | 0.006 |
| Enterococcus | 5.488 | 0.158 | 0.029 | 0.000 | 0.006 |
| Phascolarctobacterium | 0.971 | 2.937 | 3.026 | 0.007 | 0.062 |
| Ruminococcus_gnavus_group | 0.698 | 1.654 | 2.371 | 0.027 | 0.161 |
| Lachnoclostridium | 0.375 | 1.040 | 2.773 | 0.001 | 0.012 |
| Weissella | 1.253 | 0.073 | 0.059 | 0.015 | 0.113 |
| Parasutterella | 0.142 | 0.575 | 4.061 | 0.000 | 0.002 |
| Desulfovibrio | 0.557 | 0.113 | 0.203 | 0.032 | 0.161 |
| Haemophilus | 0.228 | 0.346 | 1.517 | 0.043 | 0.183 |
| Fusobacterium | 0.023 | 0.540 | 22.963 | 0.030 | 0.161 |
| Lachnospira | 0.428 | 0.116 | 0.270 | 0.013 | 0.111 |
| Catenibacterium | 0.069 | 0.417 | 6.042 | 0.000 | 0.008 |
| Pediococcus | 0.030 | 0.420 | 13.828 | 0.004 | 0.046 |
| Eubacterium_ruminantium | 0.256 | 0.106 | 0.415 | 0.040 | 0.175 |
| Methanosphaera | 0.325 | 0.001 | 0.002 | 0.006 | 0.059 |
| Prevotellaceae_NK3B31_group | 0.002 | 0.274 | 116.650 | 0.000 | 0.002 |
| Hungatella | 0.116 | 0.097 | 0.836 | 0.029 | 0.161 |
| Aggregatibacter | 0.020 | 0.181 | 8.991 | 0.001 | 0.020 |
| Rikenellaceae_RC9_gut_group | 0.180 | 0.000 | 0.003 | 0.000 | 0.001 |
| Sutterella | 0.036 | 0.139 | 3.805 | 0.016 | 0.115 |
| Lachnospiraceae_UCG-004 | 0.103 | 0.066 | 0.646 | 0.004 | 0.046 |
| Methanobrevibacter | 0.099 | 0.000 | 0.003 | 0.000 | 0.002 |
| Eisenbergiella | 0.014 | 0.056 | 3.983 | 0.038 | 0.175 |
| Lactococcus | 0.053 | 0.007 | 0.133 | 0.024 | 0.150 |
| Acidaminococcus | 0.043 | 0.016 | 0.378 | 0.015 | 0.113 |
| Scardovia | 0.035 | 0.001 | 0.035 | 0.019 | 0.132 |
| Olsenella | 0.020 | 0.004 | 0.224 | 0.031 | 0.161 |
| Lachnospiraceae_ND3007 | 0.011 | 0.011 | 0.972 | 0.023 | 0.150 |
| Actinidia_chinensis | 0.000 | 0.020 | ＜0.001 | 0.004 | 0.046 |
| Methanocorpusculum | 0.016 | 0.000 | 0.000 | 0.040 | 0.175 |
| Gram-negative_bacterium_cTPY-13 | 0.000 | 0.011 | ＜0.001 | 0.004 | 0.046 |
| Epulopiscium | 0.010 | 0.000 | 0.000 | 0.040 | 0.175 |

| Supplementary Table 10. Differential fecal metabolites between the APCI and N-APCI groups (*P* < 0.05). | | | | | |
| --- | --- | --- | --- | --- | --- |
| Metabolites | log2FC  (APCI_ /N-APCI) | P-value | VIP | RT | m/z |
| Stercobilin | 2.403 | 0.0018 | 22.251 | 5.112 | 595.349 |
| (3r,5r,6r,7s,9s,10r,13r,17r)-17-((r)-5-methoxy-5-oxopentan-2-yl)-10,13-dimethylhexadecahydro-1h-cyclopenta[a]phenanthrene-3,6,7-triyl triacetate | 2.142 | 0.0153 | 9.449 | 3.304 | 549.343 |
| N-acetyl-l-phenylalanine | 0.683 | 0.0157 | 7.764 | 4.698 | 164.072 |
| Koumine | 2.917 | 0.0445 | 6.865 | 3.026 | 307.202 |
| Eudesmin | 1.011 | 0.0218 | 6.477 | 0.528 | 385.169 |
| 3-dehydroepiandrosterone sulfate | -0.871 | 0.0222 | 6.462 | 0.436 | 367.158 |
| Benzoic acid, 2-fluoro- | -2.177 | 0.0144 | 5.834 | 3.583 | 139.007 |
| Cysteine-s-sulfate | 0.285 | 0.0101 | 5.803 | 1.077 | 200.002 |
| L-pyroglutamic acid | 1.017 | 0.0280 | 5.521 | 5.446 | 128.035 |
| Citalopram | -0.731 | 0.0127 | 5.520 | 3.777 | 325.152 |
| Sulfobacin b | 0.948 | 0.0367 | 4.643 | 0.517 | 574.451 |
| Bioresmethrin | 2.213 | 0.0005 | 4.503 | 4.245 | 321.181 |
| Glutamic acid | 0.433 | 0.0368 | 4.454 | 7.012 | 146.046 |
| 2,2-bis[hydroxymethyl]-2,2',2''-nitrilotriethanol | 2.538 | 0.0070 | 4.208 | 3.347 | 210.112 |
| 1-oleoyl-sn-glycero-3-phosphocholine | -2.456 | 0.0378 | 4.205 | 3.183 | 556.331 |
| Arachidonoylserinol | 0.937 | 0.0046 | 4.136 | 1.018 | 376.294 |
| N-acetyl-l-glutamate | 0.904 | 0.0118 | 3.909 | 6.878 | 188.056 |
| DL-Glutamic acid | 0.383 | 0.0488 | 3.806 | 7.025 | 148.061 |
| Chloroquine | 1.834 | 0.0067 | 3.721 | 5.774 | 320.197 |
| .beta.-estradiol 3-benzoate | 3.125 | 0.0184 | 3.617 | 2.57 | 359.194 |
| .beta.-zearalenol | 2.070 | 0.0017 | 3.603 | 4.231 | 319.166 |
| Hecogenin | 0.961 | 0.0132 | 3.561 | 3.576 | 429.297 |
| Fa 18:1+3o | -1.153 | 0.0026 | 3.529 | 3.123 | 329.233 |
| Daidzein 4'-sulfate | 2.359 | 0.0470 | 3.490 | 0.478 | 333.007 |
| Tetrahydrozoline | -1.420 | 0.0496 | 3.449 | 0.955 | 201.160 |
| His-ser | -1.446 | 0.0161 | 3.429 | 1.638 | 241.083 |
| falcarinol | 1.338 | 0.0039 | 3.423 | 3.595 | 259.182 |
| Triamcinolone acetonide | -0.661 | 0.0271 | 3.393 | 3.777 | 357.178 |
| Trans-4-(aminomethyl)cyclohexanecarboxylic acid | 1.923 | 0.0216 | 3.391 | 0.842 | 158.118 |
| P-coumaryl alcohol | -2.260 | 0.0284 | 3.329 | 5.366 | 149.046 |
| Lauroyl-l-carnitine | 1.187 | 0.0099 | 3.064 | 2.657 | 342.265 |
| (-)-.alpha.-kainic acid | 3.741 | 0.0291 | 3.009 | 6.79 | 196.097 |
| Linoleoylcarnitine | -1.871 | 0.0110 | 2.979 | 3.444 | 424.342 |
| Aloeemodin | -2.199 | 0.0324 | 2.959 | 0.503 | 607.291 |
| Lysine | 0.632 | 0.0324 | 2.883 | 9.029 | 145.098 |
| 2-cis-4-trans-abscisic acid | -1.039 | 0.0241 | 2.866 | 0.549 | 287.139 |
| Trans-traumatic acid | -1.263 | 0.0344 | 2.805 | 4.689 | 227.140 |
| Allyl isothiocyanate | 0.532 | 0.0186 | 2.798 | 3.399 | 98.025 |
| Octhilinone | 1.448 | 0.0370 | 2.770 | 0.733 | 214.144 |
| Paraoxon | 2.208 | 0.0418 | 2.767 | 1.18 | 274.039 |
| Strychnine | 2.235 | 0.0164 | 2.696 | 3.365 | 335.196 |
| L-pipecolic acid | 0.558 | 0.0386 | 2.665 | 9.042 | 130.086 |
| Madecassic acid | 2.208 | 0.0037 | 2.649 | 5.102 | 549.344 |
| 4-oxo-4-(3-pyridyl)butanal | 1.450 | 0.0013 | 2.455 | 4.731 | 164.071 |
| Trp-His-Lys | 2.193 | 0.0029 | 2.403 | 5.14 | 470.264 |
| (2r,3r,4r,5r,6s)-2-[[(2r,3s,4s,5r,6r)-6-[1,7-bis(4-hydroxyphenyl)heptan-3-yloxy]-3,4,5-trihydroxyoxan-2-yl]methoxy]-6-methyloxane-3,4,5-triol | -1.626 | 0.0285 | 2.391 | 3.066 | 607.385 |
| Ferulate | -1.248 | 0.0396 | 2.299 | 2.999 | 193.051 |
| 7-methylguanine | 0.317 | 0.0019 | 2.234 | 1.041 | 164.002 |
| 2,3-dinor-8-isoprostaglandin-f2.alpha. | 1.753 | 0.0325 | 2.202 | 0.374 | 651.430 |
| Benzamide, n-[5-[2-(3,5-dimethoxyphenyl)ethyl]-1h-pyrazol-3-yl]-4-[(3r,5s)-3,5-dimethyl-1-piperazinyl]-, rel- | 1.333 | 0.0246 | 2.177 | 6.669 | 486.271 |
| 1,2,4-triazin-5-amine, n-([1,1'-biphenyl]-4-ylmethyl)-6-phenyl-3-(2-pyridinyl)- | -0.616 | 0.0348 | 2.163 | 1.698 | 416.197 |
| Gentisic acid | -1.093 | 0.0310 | 2.162 | 1.174 | 153.020 |
| Resveratrol | -1.031 | 0.0391 | 2.156 | 1.92 | 227.067 |
| Stearamide | -1.170 | 0.0471 | 2.123 | 0.601 | 306.279 |
| Prednisone | 1.604 | 0.0035 | 2.120 | 3.631 | 359.194 |
| N-palmitoyl-d-sphingosine | -0.962 | 0.0218 | 2.076 | 0.554 | 520.509 |
| Endothal | -2.011 | 0.0461 | 2.067 | 0.79 | 185.038 |
| Ginkgolide b | 1.503 | 0.0488 | 2.030 | 2.428 | 407.137 |
| O-t-butyl-l-serine, methyl ester | 1.772 | 0.0152 | 2.025 | 6.497 | 176.128 |
| Acenapthylene | -1.259 | 0.0433 | 2.015 | 4.237 | 152.056 |
| 5alpha-androstan-17beta-ol-3-one | -1.643 | 0.0217 | 1.998 | 0.856 | 215.176 |
| Lpc 16:0 | -1.309 | 0.0277 | 1.996 | 1.896 | 540.337 |
| 2-linoleoyl-1-palmitoyl-sn-glycero-3-phosphoethanolamine | -1.612 | 0.0235 | 1.996 | 0.968 | 714.508 |
| 1-pentanone, 1-(4-hydroxy-3-methoxyphenyl)-2-(1-pyrrolidinyl)- | 1.901 | 0.0017 | 1.952 | 4.776 | 261.161 |
| Hygromycin b | 1.354 | 0.0433 | 1.854 | 2.099 | 352.139 |
| (2e,4e)-n-[2-(4-hydroxyphenyl)ethyl]dodeca-2,4-dienamide | 0.953 | 0.0154 | 1.783 | 3.06 | 314.234 |
| Diethyl azelate | 2.287 | 0.0263 | 1.779 | 2.543 | 245.165 |
| Fingolimod | 0.428 | ＜0.001 | 1.703 | 1.722 | 290.269 |
| 6-quinoxalinecarbonitrile, 1,2,3,4-tetrahydro-7-nitro-2,3-dioxo- | -1.445 | 0.0125 | 1.661 | 0.458 | 203.002 |
| Racemoramide | 1.805 | 0.0058 | 1.641 | 5.293 | 306.181 |
| 4-thiazolidinecarboxylic acid, 2-undecyl-, (4r)- | -0.740 | 0.0181 | 1.626 | 4.664 | 286.177 |
| Val-Ser-Arg | 0.688 | 0.0401 | 1.608 | 6.059 | 181.097 |
| Tetramethrin | 1.305 | 0.0396 | 1.559 | 2.635 | 314.160 |
| Hydromorphone | -1.749 | 0.0209 | 1.515 | 7.21 | 286.140 |
| Pomiferin | -2.154 | 0.0249 | 1.482 | 3.134 | 419.171 |
| Lithocholylglycine | 1.879 | 0.0055 | 1.480 | 3.338 | 432.312 |
| Ochrephilone | 1.678 | 0.0400 | 1.451 | 3.508 | 381.192 |
| (-)-riboflavin | 0.603 | 0.0345 | 1.443 | 4.017 | 377.146 |
| Isradipine | 1.123 | 0.0355 | 1.428 | 1.376 | 394.150 |
| Myristicine | 1.561 | 0.0161 | 1.400 | 3.151 | 193.088 |
| Imazethapyr | -1.068 | 0.0166 | 1.379 | 5.391 | 288.157 |
| Prostaglandin e2 | 1.253 | 0.0495 | 1.362 | 1.336 | 370.277 |
| D-Tagatose | -1.026 | 0.0485 | 1.360 | 4.799 | 179.056 |
| 1,3-propanediol, 2-amino-2-[2-(3-azido-4-octylphenyl)ethyl]- | 0.932 | 0.0366 | 1.351 | 0.601 | 349.274 |
| [3-hydroxy-4-[(2s,3r,4s,5s,6r)-3,4,5-trihydroxy-6-(hydroxymethyl)oxan-2-yl]oxyphenyl]methyl 3,4-dihydroxybenzoate | 1.280 | 0.0473 | 1.345 | 0.915 | 437.091 |
| Ascorbyl stearate | 1.362 | 0.0098 | 1.339 | 5.126 | 441.286 |
| Withaferin a | 2.182 | 0.0019 | 1.324 | 5.106 | 471.268 |
| Octyl gallate | -1.254 | 0.0324 | 1.315 | 0.46 | 281.132 |
| Perifosine | 2.086 | 0.0438 | 1.282 | 6.759 | 210.112 |
| Leptomycin b | 1.561 | 0.0097 | 1.267 | 4.802 | 539.359 |
| Repaglinide | 0.852 | 0.0225 | 1.266 | 3.62 | 453.294 |
| N-glycolylneuraminic acid | -1.576 | 0.0272 | 1.258 | 2.678 | 308.113 |
| Trp-Val | -0.801 | 0.0222 | 1.253 | 4.721 | 302.172 |
| Psoromic acid | -2.072 | 0.0144 | 1.235 | 5.303 | 357.076 |
| 4-mercaptobenzoic acid | -0.905 | 0.0291 | 1.227 | 1.621 | 152.986 |
| Cytisine | -1.915 | 0.0159 | 1.218 | 4.16 | 191.118 |
| Paeonolide | 0.733 | 0.0423 | 1.199 | 0.77 | 459.169 |
| 7-methyluric acid | 1.168 | 0.0089 | 1.184 | 4.118 | 181.018 |
| N6-(1-iminoethyl)-l-lysine | 1.358 | 0.0129 | 1.177 | 1.851 | 210.112 |
| Nateglinide | -1.004 | 0.0069 | 1.153 | 4.419 | 316.188 |
| 2-(2h-benzotriazol-2-yl)-4-(1,1,3,3-tetramethylbutyl)phenol | 1.461 | 0.0065 | 1.126 | 5.388 | 322.177 |
| Bestatin | 1.903 | 0.0009 | 1.125 | 4.721 | 307.166 |
| Colupone isomer (mse) | 1.250 | 0.0286 | 1.120 | 4.865 | 467.301 |
| Xylitol | -1.031 | 0.0255 | 1.106 | 2.183 | 151.088 |
| NCGC00347708-02 | 0.690 | 0.0204 | 1.104 | 0.476 | 709.435 |
| Cuminaldehyde | -0.661 | 0.0310 | 1.100 | 11.9 | 149.083 |
| Sebacic acid | 0.909 | 0.0318 | 1.063 | 5.913 | 201.113 |
| 2-phenylacetamide | 1.050 | 0.0008 | 1.048 | 4.287 | 136.076 |
| 3-acetyl-11-keto-.beta.-boswellic acid | 1.109 | 0.0395 | 1.039 | 4.847 | 511.328 |
| 3-hydroxyspirost-9(11)-en-12-one, 3-ac | 1.041 | 0.0108 | 1.030 | 2.584 | 471.309 |
| Oleana-1,9(11)-dien-28-oic acid, 2-cyano-3,12-dioxo-, methyl ester | -1.611 | 0.0378 | 1.008 | 1.985 | 506.330 |
| Tulipinolide | -1.134 | 0.0083 | 1.002 | 2.364 | 289.156 |
| (cis+trans)-nerodilol | 0.337 | 0.0121 | 1.000 | 0.557 | 109.101 |

| Supplementary Table 11. Differential fecal metabolites between the S-APCI and NS-NAPCI groups (*P* < 0.05). | | | | |
| --- | --- | --- | --- | --- |
| Metabolites | log2FC  (S-APCI /  NS-NAPCI) | P-value | RT | m/z |
| Stercobilin | -2.446 | 0.0149 | 5.112 | 595.349 |
| N-acetyl-l-phenylalanine | -0.988 | 0.0081 | 4.698 | 164.072 |
| Benzoic acid, 2-fluoro- | 2.460 | 0.0247 | 3.583 | 139.007 |
| Cysteine-s-sulfate | -0.284 | 0.0106 | 1.077 | 200.002 |
| Piperidine | -0.618 | 0.0375 | 5.006 | 86.096 |
| Dl-a-hydroxybutyric acid | -1.186 | 0.0435 | 3.555 | 103.040 |
| Trans-2,3-dimethylacrylic acid | 1.038 | 0.0191 | 1.153 | 99.009 |
| Arachidonoylserinol | -1.002 | 0.0123 | 1.018 | 376.294 |
| Fa 18:1+3o | 1.377 | 0.0045 | 3.123 | 329.233 |
| 2.2,2-bis[hydroxymethyl]-2,2',2''-nitrilotriethanol | -2.855 | 0.0188 | 3.347 | 210.112 |
| N-acetyl-l-aspartic acid | -0.958 | 0.0137 | 6.988 | 174.041 |
| Aloeemodin | 2.261 | 0.0313 | 0.503 | 607.291 |
| Hecogenin | -0.975 | 0.0469 | 3.576 | 429.297 |
| Myristic acid | -1.306 | 0.0242 | 0.723 | 227.202 |
| .beta.-estradiol 3-benzoate | -3.442 | 0.0359 | 2.570 | 359.194 |
| Bioresmethrin | -1.878 | 0.0172 | 4.245 | 321.181 |
| Lysine | -0.854 | 0.0181 | 9.029 | 145.098 |
| L-dihydroorotate | 0.979 | 0.0331 | 7.932 | 157.014 |
| L-aspartic acid | -0.987 | 0.0357 | 7.100 | 132.030 |
| Dodecanedioic acid | 1.691 | 0.0497 | 5.311 | 229.145 |
| His-ser | 1.515 | 0.0238 | 1.638 | 241.083 |
| Chloroquine | -1.804 | 0.0366 | 5.774 | 320.197 |
| .alpha.-keto-.gamma.-(methylthio)butyric acid | 1.038 | 0.0191 | 1.151 | 147.012 |
| Falcarindiol | -1.445 | 0.0170 | 3.595 | 259.182 |
| L-pipecolic acid | -0.666 | 0.0493 | 9.042 | 130.086 |
| .beta.-zearalenol | -1.842 | 0.0244 | 4.231 | 319.166 |
| 2-cis-4-trans-abscisic acid | 1.209 | 0.0497 | 0.549 | 287.139 |
| S-indoxacarb | 0.872 | 0.0055 | 2.843 | 249.028 |
| Ferulate | 1.516 | 0.0345 | 2.999 | 193.051 |
| Strychnine | -2.239 | 0.0459 | 3.365 | 335.196 |
| 1,2,4-triazin-5-amine, n-([1,1'-biphenyl]-4-ylmethyl)-6-phenyl-3-(2-pyridinyl)- | 0.754 | 0.0311 | 1.698 | 416.197 |
| Bullatine a | -2.025 | 0.0381 | 1.426 | 344.262 |
| Madecassic acid | -2.523 | 0.0218 | 5.102 | 549.344 |
| Salicylaldehyde | -0.813 | 0.0456 | 5.685 | 123.055 |
| Stearamide | 1.193 | 0.0385 | 0.601 | 306.279 |
| Prostaglandin e2 | -2.125 | 0.0103 | 1.336 | 370.277 |
| Man5 | 1.238 | 0.0339 | 4.611 | 629.197 |
| Trp-His-Lys | -2.225 | 0.0196 | 5.140 | 470.264 |
| Oxymatrine | -1.803 | 0.0403 | 3.017 | 247.180 |
| Resveratrol | 1.130 | 0.0455 | 1.920 | 227.067 |
| Qingyangshengenin | 1.133 | 0.0359 | 3.370 | 345.217 |
| 7-methylguanine | -0.268 | 0.0393 | 1.041 | 164.002 |
| Glufosinate | -0.950 | 0.0498 | 3.861 | 180.053 |
| Prednisone | -1.507 | 0.0248 | 3.631 | 359.194 |
| 4-thiazolidinecarboxylic acid, 2-undecyl-, (4r)- | 0.925 | 0.0128 | 4.664 | 286.177 |
| 4-oxo-4-(3-pyridyl)butanal | -1.182 | 0.0380 | 4.731 | 164.071 |
| 2-hexenal | 0.810 | 0.0233 | 1.721 | 99.056 |
| 6-quinoxalinecarbonitrile, 1,2,3,4-tetrahydro-7-nitro-2,3-dioxo- | 1.982 | 0.0183 | 0.458 | 203.002 |
| N6-(1-iminoethyl)-l-lysine | -1.875 | 0.0122 | 1.851 | 210.112 |
| 21-hydroxyprogesterone | 1.040 | 0.0268 | 2.352 | 329.233 |
| Lithocholylglycine | -2.222 | 0.0248 | 3.338 | 432.312 |
| Octyl gallate | 1.500 | 0.0256 | 0.460 | 281.132 |
| 4.4,15,26-trihydroxy-1-oxa-4,9,15,20,26,31-hexazacyclotritriacontane-5,8,16,19,27,30-hexone | -2.528 | 0.0492 | 0.797 | 601.305 |
| Pomiferin | 2.187 | 0.0366 | 3.134 | 419.171 |
| 1-pentanone, 1-(4-hydroxy-3-methoxyphenyl)-2-(1-pyrrolidinyl)- | -1.833 | 0.0237 | 4.776 | 261.161 |
| 2'-hydroxy-4-methoxychalcone | 1.685 | 0.0307 | 2.807 | 253.108 |
| Xylitol | 1.551 | 0.0033 | 2.183 | 151.088 |
| N-glycolylneuraminic acid | 1.683 | 0.0377 | 2.678 | 308.113 |
| Cytisine | 2.163 | 0.0158 | 4.160 | 191.118 |
| Ochrephilone | -1.752 | 0.0195 | 3.508 | 381.192 |
| Fingolimod | -0.329 | 0.0035 | 1.722 | 290.269 |
| Phosphatidylethanolamine lyso alkenyl 18 | -1.553 | 0.0306 | 3.771 | 462.322 |
| Trp-Val | 0.904 | 0.0376 | 4.721 | 302.172 |
| Imazethapyr | 1.100 | 0.0401 | 5.391 | 288.157 |
| Methasterone | -1.799 | 0.0185 | 0.511 | 283.243 |
| Withaferin a | -2.313 | 0.0144 | 5.106 | 471.268 |
| Nateglinide | 1.079 | 0.0146 | 4.419 | 316.188 |
| Cys-Gln | 0.825 | 0.0336 | 1.391 | 248.093 |
| Cuminaldehyde | 0.995 | 0.0169 | 11.900 | 149.083 |
| Myristicine | -1.979 | 0.0132 | 3.151 | 193.088 |
| Orotate | 1.622 | 0.0162 | 4.703 | 155.010 |
| 7-methyluric acid | -1.170 | 0.0389 | 4.118 | 181.018 |
| Ala-Val-Lys | 1.224 | 0.0445 | 7.003 | 317.218 |
| 2-(2h-benzotriazol-2-yl)-4-(1,1,3,3-tetramethylbutyl)phenol | -1.661 | 0.0215 | 5.388 | 322.177 |
| Val-Gly-Val | 1.087 | 0.0090 | 4.990 | 272.162 |
| Gabapentin | -0.846 | ＜0.001 | 0.663 | 172.133 |
| Pyrimidine | -0.748 | 0.0369 | 5.685 | 81.045 |
| 7-demethylsuberosin | -1.158 | 0.0375 | 7.454 | 231.098 |

| Supplementary Table 12. KEGG pathway enrichment analysis of differential metabolites between the APCI and N-APCI groups. | | | |
| --- | --- | --- | --- |
| Pathway | N-APCI-vs-APCI (33) | All (732) | P-value |
| AGE-RAGE signaling pathway in diabetic complications | 1 | 2 | 0.088 |
| Cyanoamino acid metabolism | 2 | 12 | 0.098 |
| Benzoate degradation | 1 | 4 | 0.169 |
| Fluorobenzoate degradation | 1 | 4 | 0.169 |
| Xylene degradation | 1 | 4 | 0.169 |
| Styrene degradation | 1 | 4 | 0.169 |
| Riboflavin metabolism | 1 | 4 | 0.169 |
| Phenylalanine metabolism | 2 | 18 | 0.193 |
| Sesquiterpenoid and triterpenoid biosynthesis | 1 | 5 | 0.206 |
| Biosynthesis of alkaloids derived from ornithine, lysine and nicotinic acid | 2 | 19 | 0.209 |
| alpha-Linolenic acid metabolism | 1 | 6 | 0.243 |
| Arachidonic acid metabolism | 2 | 21 | 0.244 |
| Biosynthesis of terpenoids and steroids | 2 | 22 | 0.261 |
| Arginine biosynthesis | 1 | 7 | 0.277 |
| Naphthalene degradation | 1 | 7 | 0.277 |
| Biosynthesis of phenylpropanoids | 3 | 45 | 0.331 |
| Sphingolipid metabolism | 1 | 9 | 0.341 |
| Carotenoid biosynthesis | 1 | 9 | 0.341 |
| Insulin resistance | 1 | 9 | 0.341 |
| Degradation of aromatic compounds | 2 | 28 | 0.363 |
| Caffeine metabolism | 1 | 10 | 0.371 |
| Pentose and glucuronate interconversions | 1 | 12 | 0.428 |
| Glutathione metabolism | 1 | 12 | 0.428 |
| Porphyrin metabolism | 1 | 14 | 0.479 |
| Lysine degradation | 1 | 15 | 0.503 |
| Galactose metabolism | 1 | 16 | 0.526 |
| Biosynthesis of amino acids | 2 | 40 | 0.550 |
| Aminobenzoate degradation | 1 | 18 | 0.568 |
| Nicotinate and nicotinamide metabolism | 1 | 18 | 0.568 |
| Biosynthesis of plant secondary metabolites | 3 | 66 | 0.587 |
| Microbial metabolism in diverse environments | 6 | 135 | 0.589 |
| Cysteine and methionine metabolism | 1 | 21 | 0.626 |
| 2-Oxocarboxylic acid metabolism | 1 | 22 | 0.643 |
| Pyrimidine metabolism | 1 | 23 | 0.660 |
| Metabolic pathways | 23 | 526 | 0.691 |
| Tyrosine metabolism | 1 | 26 | 0.705 |
| Biosynthesis of plant hormones | 1 | 26 | 0.705 |
| Amino sugar and nucleotide sugar metabolism | 1 | 28 | 0.732 |
| ABC transporters | 2 | 59 | 0.764 |
| Biosynthesis of secondary metabolites | 10 | 274 | 0.854 |
| Biosynthesis of alkaloids derived from shikimate pathway | 1 | 41 | 0.857 |
