# Supplementary material for: Sarcopenia Predicts Postoperative Cognitive Impairment and Poor Surgical Outcomes in Older Adults: A Prospective Cohort Study
Source: J Cachexia Sarcopenia Muscle. 2026 Jul 15;17(4):e70346. doi: 10.1002/jcsm.70346 (PMC13372572; doi:10.1002/jcsm.70346)
Supplement: Supplementary file 2 — Figure S1: Paired MMSE Scores Preoperation and Postoperation (n = 443).The plot shows paired preoperative (Pre‐MMSE) and postoperative (Post‐MMSE) Mini‐Mental State Examination (MMSE) scores for 443 patients. Blue points represent preoperative MMSE scores, while red points represent postoperative MMSE scores. Black lines indicate cases where postoperative scores decreased compared with preoperative scores, while grey lines indicate cases where postoperative scores remained the same or increased. The size of each point reflects the repeat count, indicating the number of occurrences of specific MMSE scores across patients. Figure S2: Flow chart of the study. [file JCSM-17-e70346-s002.docx]

Supplementary Figures


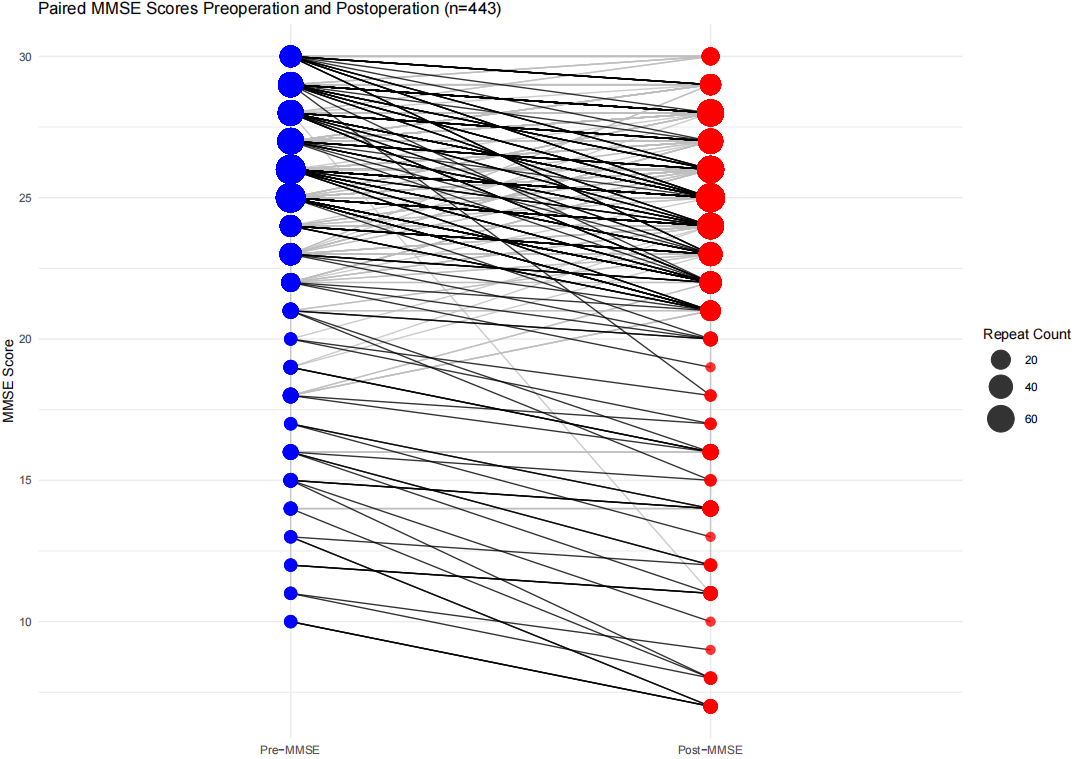


Supplementary figure 1: Paired MMSE Scores Preoperation and Postoperation (n=443).

The plot shows paired preoperative (Pre-MMSE) and postoperative (Post-MMSE) Mini-Mental State Examination (MMSE) scores for 443 patients. Blue points represent preoperative MMSE scores, while red points represent postoperative MMSE scores. Black lines indicate cases where postoperative scores decreased compared to preoperative scores, while gray lines indicate cases where postoperative scores remained the same or increased. The size of each point reflects the repeat count, indicating the number of occurrences of specific MMSE scores across patients.


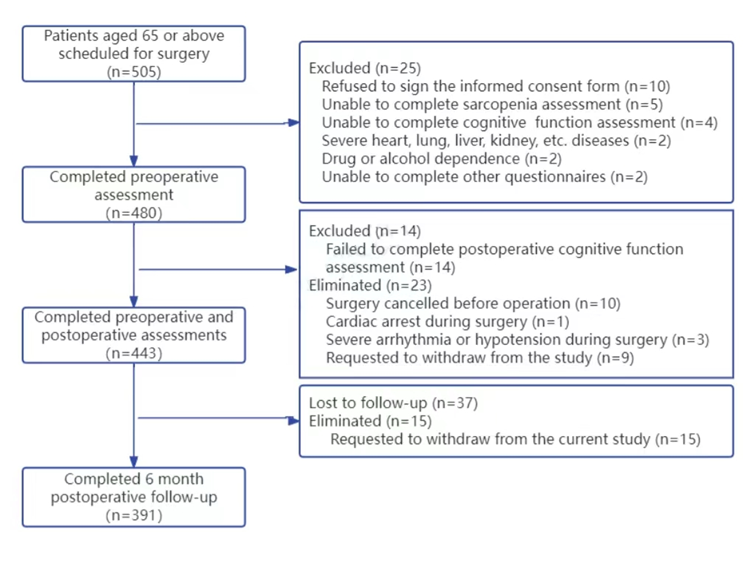


Supplementary figure 2. Flow chart of the study
